# Allostery in the nitric oxide dioxygenase mechanism of flavohemoglobin

**DOI:** 10.1074/jbc.RA120.016637

**Published:** 2020-12-17

**Authors:** Anne M. Gardner, Paul R. Gardner

**Affiliations:** 1 Research and Development Division, Miami Valley Biotech, Dayton, Ohio, USA; 2 Division of Critical Care Medicine, Cincinnati Children's Hospital Medical Center, Cincinnati, Ohio, USA; 3 Chemistry and Biochemistry Department, University of Dayton, Dayton, Ohio, USA

**Keywords:** flavohemoglobin, nitric oxide dioxygenase, hemoglobin, myoglobin, nitric oxide, oxygen, allostery, flavin, heme, electron transfer, BSA, bovine serum albumin, Cygb, cytoglobin, ET, electron transfer, flavoHb, flavohemoglobin, Hb, hemoglobin, LT, long tunnel, Mb, myoglobin, Ngb, neuroglobin, ST, short tunnel, TMAO, trimethylamine-*N*-oxide, τ_T_, transition time, Φ_dis_, photodissociation, WT, wild-type

## Abstract

The substrates O_2_ and NO cooperatively activate the NO dioxygenase function of *Escherichia coli* flavohemoglobin. Steady-state and transient kinetic measurements support a structure-based mechanistic model in which O_2_ and NO movements and conserved amino acids at the E11, G8, E2, E7, B10, and F7 positions within the globin domain control activation. In the cooperative and allosteric mechanism, O_2_ migrates to the catalytic heme site *via* a long hydrophobic tunnel and displaces LeuE11 away from the ferric iron, which forces open a short tunnel to the catalytic site gated by the ValG8/IleE15 pair and LeuE11. NO permeates this tunnel and leverages upon the gating side chains triggering the CD loop to furl, which moves the E and F-helices and switches an electron transfer gate formed by LysF7, GlnE7, and water. This allows FADH_2_ to reduce the ferric iron, which forms the stable ferric–superoxide–TyrB10/GlnE7 complex. This complex reacts with internalized NO with a bimolecular rate constant of 10^10^ M^−1^ s^−1^ forming nitrate, which migrates to the CD loop and unfurls the spring-like structure. To restart the cycle, LeuE11 toggles back to the ferric iron. Actuating electron transfer with O_2_ and NO movements averts irreversible NO poisoning and reductive inactivation of the enzyme. Together, structure snapshots and kinetic constants provide glimpses of intermediate conformational states, time scales for motion, and associated energies.

Distantly related members of the Hb superfamily, including flavoHb, HbN, phytoglobin, Mb, and Cygb, function as NO dioxygenases (NODs) (EC 1.14.12.17) to limit NO toxicity and control NO signaling in cells (1, 2). A greater understanding of the enzyme mechanism promises insights for the design of novel antibiotics, vasorelaxants, and antitumor agents. We previously suggested an ordered ping-pong mechanism in which O_2_ enters the distal pocket and binds the ferrous heme forming a stable ferric superoxide intermediate that reacts with NO to form NO_3_^−^ and Fe^3+^, and in which Fe^3+^ is rapidly reduced to repeat the catalytic cycle (1, 2, 3, 4, 5, 6). In support of the mechanism, the conserved distal TyrB10 hydroxyl group in flavoHb and HbN confers high O_2_ affinity by stabilizing the proposed intermediate Fe^3+^O_2_^−^ thus allowing rapid turnover (4, 7, 8, 9). Also, the ferric heme was shown to be reduced by NADH, in the absence of O_2_ or NO, at rates approaching NOD turnover rates suggesting an unregulated reduction step (4). However, the mechanism has failed to fully account for weak and reversible NO inhibition and a large O_2_ requirement relative to NO and O_2_ affinities measured for the ferrous heme (1, 4, 5, 10). Indeed, the relatively high affinity of ferrous flavoHbs for NO (4, 5) led Hausladen *et al.* (11) to suggest a reversed order ping-pong mechanism in which a ferric nitroxyl intermediate reacts with O_2_ to form NO_3_^−^, a reaction with an intrinsically small rate constant that is inconsistent with the large NO dioxygenation rate constants of (flavo)Hbs and the critical role of O_2_ affinity in high turnover rates (1, 2). In addition, the 100-fold discrepancy between the NO migration rate to the ferrous heme as measured by transient kinetics and the calculated *V*_max_/*K*_m_(NO) or *k'*_NOD_ values for flavoHb-NODs (4, 5) has demanded clarification. Deficiencies in both of the proposed mechanisms has spurred greater efforts toward a resolution (2, 12, 13, 14, 15, 16). Nevertheless, the mechanism has remained enigmatic, and controversial, partly because of the physical and chemical similarities of the substrates O_2_ and NO, and also because of insufficient knowledge of the unique structures and dynamics supporting the mechanism, and an apparent underdetermination of kinetic data (4). A simple ping-pong mechanism also fails to fully explain the dioxygenase activity of Cygb (10, 17) or the alpha subunit of HbA in complex with its chaperone (18). Both NOD activities would also be expected to be exquisitely sensitive to irreversible poisoning by NO. In fact, the well-known rapid and tight binding of NO with the ferrous heme of erythrocyte HbA or muscle Mb, with comparable O_2_ association and NO oxidation (dioxygenation) rate constants (19, 20), has long diminished enthusiasm for an enzymatic NOD function for Mb and HbA, or its subunits (2, 21) or, for that matter, any globin.

A greater understanding of the NOD mechanism, and a solution to the paradox, is slowly emerging from a wealth of structural models, molecular dynamics simulations, and spectroscopic and kinetic studies of globins. O_2_ and NO tunnels and a nitrate egress pathway have been suggested to play roles in the NOD function of the truncated HbN from *Mycobacterium tuberculosis* (22, 23). A long hydrophobic tunnel that runs parallel to the H helix and perpendicular to the heme plane is thought to allow NO (22, 24, 25), or O_2_ (16, 24, 26), to access the distal heme reaction chamber, while a short tunnel formed between the G and H helices at the AlaG5 and LeuH8 residues allows O_2_ (22), or NO (24, 26, 27), to access the distal heme pocket. Moreover, movement of PheE15 caused by O_2_ binding to the ferrous heme and TyrB10-GlnE11 hydrogen-bonding interactions (28) may function as a gate, in cooperation with LeuG8, that controls NO access to the heme (22, 25, 29, 30, 31). In support, a TrpE15 variant showed two- to threefold less NOD activity when reductively coupled and assayed within *E. coli* (30) but inexplicably showed no effect on NO dioxygenation by HbNFe^3+^O_2_^−^ (32) and little apparent effect on O_2_ or NO migration to the ferrous heme (30). On the other hand, replacing LeuG8 with Phe or Trp caused large effects on CO rebinding kinetics (33), suggesting a preferential pathway for CO migration. Molecular dynamics simulations of HbN map a probable escape route for NO_3_^−^ at the ThrE2 residue in the CD loop (34).

An important mechanistic clue has also been provided by studies of the mammalian Cygb-NOD. We and others have reported 10- to 100-fold larger rate constants for the ascorbate and cytochrome *b*_*5*_-mediated reduction of the ferric Cygb during NOD catalysis (10, 17). The data are explained if O_2_ and NO movements or reaction within Cygb serve as a trigger for electron transfer during catalysis (10). Evidence of an electron transfer trigger and switch would also explain how the reduction step limits the NOD function (4, 35). Furthermore, Cygb structures reveal potential tunnels and gates (36, 37) that appear functionally similar to those found in HbN.

We have observed, and now report, cooperative activation of the *E. coli* flavoHb-NOD activity by O_2_ and NO. To understand the apparent allostery, we have analyzed structures of flavoHbs, and related globins, formulated a structural and kinetic model for the NOD mechanism, and evaluated the model for its compatibility with the steady-state and transient kinetic behavior of wild-type (WT) and variant flavoHbs. The data and model allow us to derive kinetic constants and energies for a flavoHb-NOD mechanism that employs O_2_ (38) and NO-specific supply tunnels, gates, or channels for regulating NO entry to and nitrate efflux from the heme reaction center, a structure-sensitive electron transfer switch (38, 39, 40), an actuating lever to control the gates and switch, and an elastic torsion spring to store energy released by the NO dioxygenation reaction to drive and control motions. A lever and switch that together synchronize heme reduction with O_2_ and NO positioning, and thus prevent Fe^2+^NO formation, may explain the puzzling resistance of the NOD activity to irreversible NO inhibition, the observed reversible NO inhibition, and the higher-than-expected O_2_ dependence in the steady state (4, 5).

## Results

### O_2_ and NO dependence of flavoHb-NOD activity

**Figure 1:**
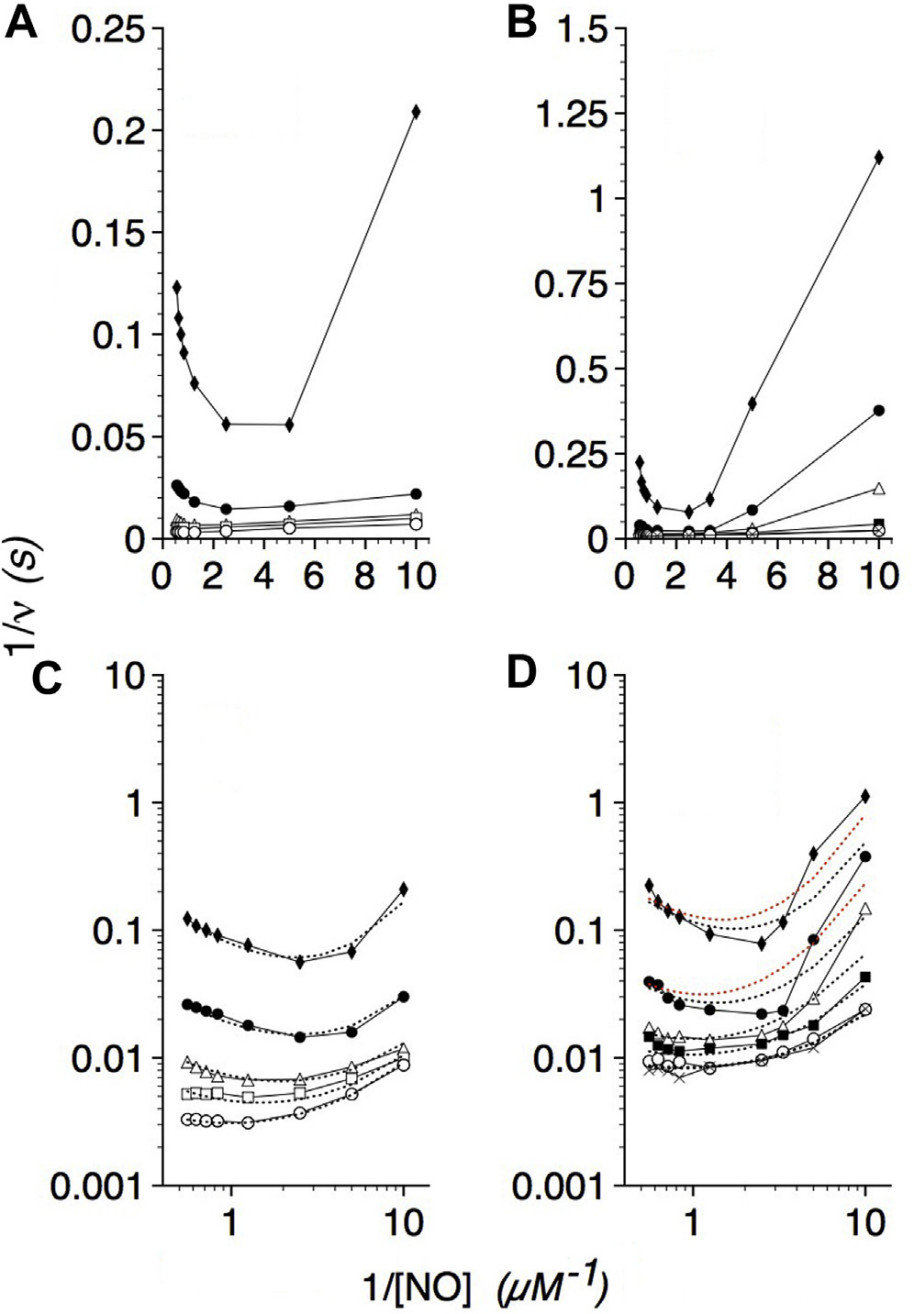
**O**_**2**_**and NO dependence of the NOD activity.***A*, Lineweaver–Burk plots of WT NOD activity at 37 °C for various NO concentrations in the presence of 8 (◆), 30 (●), 90 (Δ), 200 (□), and 670 (O) μM O_2_. *B*, Lineweaver–Burk plots of the WT NOD activity at 20 °C for various NO concentrations at 8 (◆), 30 (●), 90 (Δ), 260 (▪), 670 (O), and 1140 (X) μM O_2_. Note the 6-fold y-axis scale difference between panels *A* and *B*. *C* and *D*, log–log plots of the data from panels *A* and *B*, respectively. The kinetic constants used to fit the kinetic model with the data (*dashed lines*) are given in Table 1.

The *E. coli* flavoHb-NOD activity (4), similar to other NOD activities (5, 10, 17, 21), shows parallel lines in inverse plots of velocity *versus* [NO] with varying [O_2_] (Fig. 1*A*) supporting a ping-pong mechanism. At higher NO concentrations, NO reversibly inhibits the activity, and NO inhibition appears competitive with respect to O_2_ with roughly 50% inhibition observed at a 1:100 ratio of NO to O_2_ as previously reported (4, 5). O_2_ shows no competitive inhibition with respect to NO at O_2_:NO ratios exceeding 10,000:1. However, flavoHb shows less activity than expected with low [NO] and [O_2_] thus revealing activation by NO and O_2_. The activating effects of NO and O_2_ are more apparent at 20 °C (Fig. 1*B*). The data suggest a more complex ping-pong mechanism in which O_2_ and NO cooperatively activate NO dioxygenation.

### Structural features and a proposed NOD mechanism

**Figure 2:**
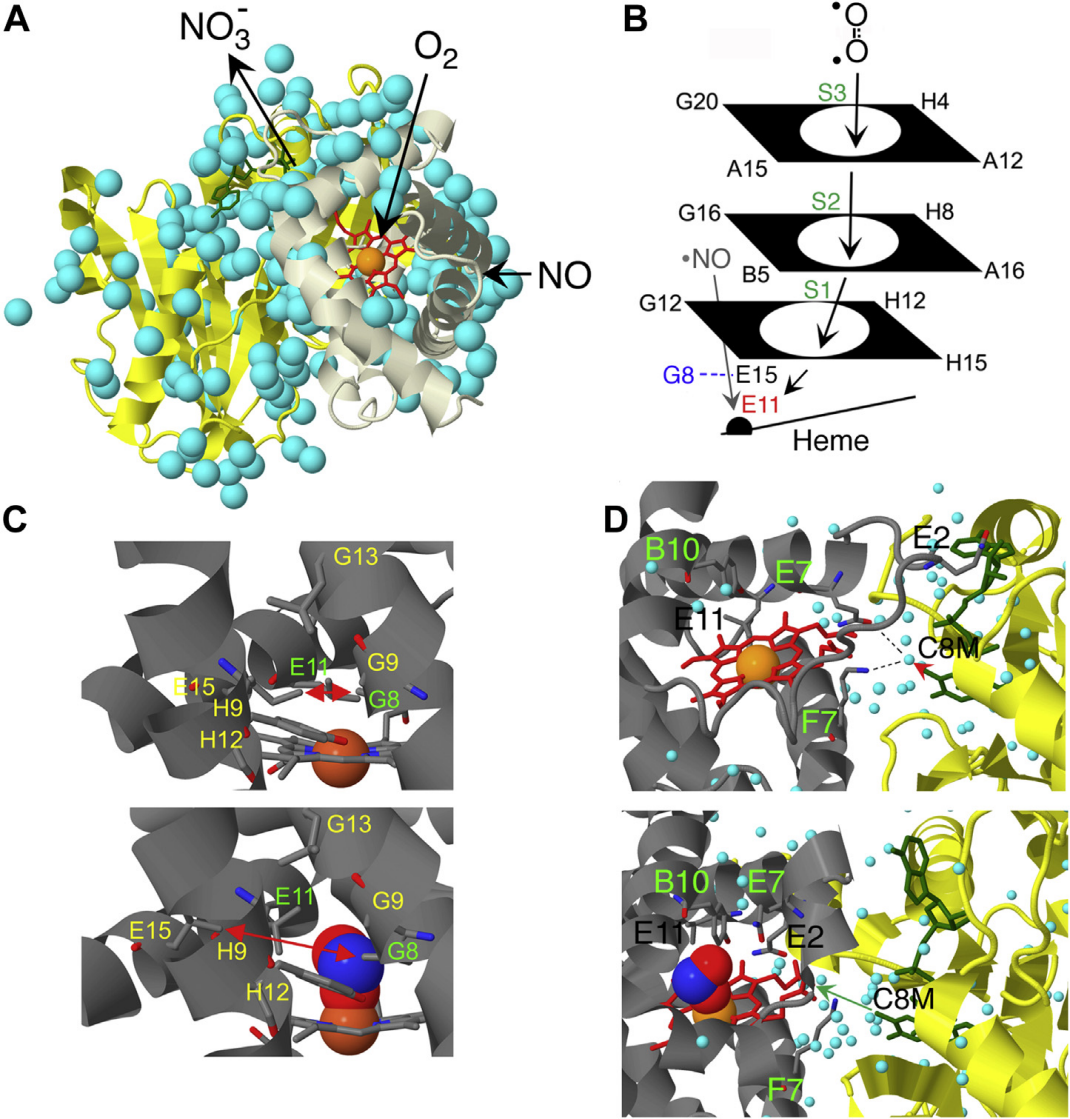
**FlavoHb structural features and the NOD mechanism.***A*, *E. coli* NOD structure (Protein Data Bank [PDB] ID: 1GVH) (43) showing proposed pathways for O_2_ and NO entry and NO_3_^−^ efflux. *B*, LT for O_2_ entry bounded by the G and H helices and traversing three strictures each formed by four side chains with the positions given. LT strictures and sites for O_2_ docking are labeled S3, S2, and S1. *C*, ST for NO entry with a narrow closed and a wide open gate (*red arrows*) as observed in the respective ferric *E. coli* NOD (PDB ID: 1GVH) (43) (*top*) and Fe^3+^NO_2_^−^*S. cerevisiae* NOD (PDB ID: 4G1V) (41) (*bottom*) structures without bound water molecules. *D*, the proposed trigger mechanism for heme reduction in *off* and *on* states as seen in the respective ferric *E. coli* NOD (*top*) and Fe^3+^NO_2_^−^*S. cerevisiae* NOD (*bottom*) structures. Residues 191 to 206 have been deleted from the foreground. The color code is Hb domain (*beige* or *gray*), reductase domain (*yellow*), heme (*red* or Corey, Pauling, Koltun [CPK] colors), iron (*orange*), FAD (*green*), water (*aqua*), and NO_2_^−^ and side chains (CPK colors).

Structural dynamics may explain the cooperative activation of NODs by O_2_ and NO, the weak reversible competitive inhibition by NO, and the larger-than-expected requirements for O_2_. Similar to the *M. tuberculosis* HbN-NOD, the flavoHb-NOD appears to utilize a hydrophobic long tunnel (LT) for O_2_ entry (38), a hydrophobic short tunnel (ST) with an amphipathic entry for NO, and a gate for NO_3_^−^ efflux (Fig. 2*A*). Six residues from the G and H helices form the outer wall of the ∼20-Å LT with three residues from the A helix and single residues from the B, E, and H helices forming the inner wall (Fig. 2*B*) (38). The passageway follows along the G-helix dipole in the positive direction. The side chains lining the LT are highly conserved among flavoHbs and single-domain Hbs (Table S1). Furthermore, the residues form a strictured or segmented tunnel with the capacity to hold up to three O_2_ molecules (38). The LT strictures show dimensions compatible with the passage of the nonpolar 3.2 Å by 4.3 Å van der Waals' dimension O_2_ molecule as previously demonstrated using molecular dynamics simulations with an estimated maximum energy barrier of 6 kcal/mol (38). However, although the static dimensions and clearances appear similar among the flavoHb and single domain Hb structures, it is noteworthy that the strictures differ with ligand states (Table S2). For example, with nitrite bound in the *Saccharomyces cerevisiae* structure (41) the clearance between the E15 and H12 residues is 3.1 Å wider and the restrictive distance between residues A16 and G16 is 1.6 Å greater, suggesting an active mechanism for controlling O_2_ movement during turnover. The LeuE11 side chain is in van der Waal's contact with the ferric iron in the *E. coli* flavoHb structure, thus apparently hindering O_2_ access to the iron, but LeuE11 does not form part of the LT.

The putative ST shows a highly conserved outer entry defined by GlyG9, GlyH9, LeuG13, and TyrH12 (Table S3) and appears to be gated in the interior by ValG8-IleE15 and LeuE11–heme interactions (Fig. 2*C*, *top*). The *S. cerevisiae* ferric-nitrite enzyme structure shows a widening of the G8-E15 gate by 4 Å and a shift of the entire E-helix by ∼2 Å toward the putative NO entry site (compare *red arrows* in Fig. 2*C*, *top* and *bottom*, and Table S4). In the open state (*bottom*), LeuE11 is displaced toward the NO entry site to form a part of the ST interior presumably guiding NO for reaction with the bound Fe^3+^O_2_^−^. By analogy to bound nitrite, O_2_ displacement of the LeuE11 may widen the gate for passage of the slightly polar 3.2 Å by 4.1 Å van der Waals' dimension NO molecule. The ST may have a capacity for more than one NO molecule.

The ferric *E. coli* flavoHb structure shows a CD loop in an unusual random coil with the GlnE2 side chain extended forming contact with the adenine ring of FAD in the reductase domain (Fig. 2*D*, *top*). A chloride ion is found bound in an anion hole in the E helix and CD loop corner (Fig. S1). Moreover, the ArgE3 guanidinium group, the E7 amide nitrogen, and a bound water bounding the anion hole show dimensions compatible with binding the trigonal planar nitrate anion in an E2-dependent efflux mechanism, whereas, with nitrite bound to the ferric heme in the *S. cerevisiae* flavoHb structure, the GlnE2 residue is found oriented inward with the D3-E4 segment in an energetically more stable alpha helix with the ArgE3 side chain oriented outward (Fig. 2*D*, *bottom*). Displacement of the LeuE11 side chain from the iron by nitrite, and presumably by nitrate, appears to twist the E-helix and exert a right-handed torsion to furl the CD loop (Fig. S1). In the unoccupied state, and presumably with nitrate release in the NOD mechanism, large phi torsion angle changes of ∼180° occur for the D4 and E3 residues in the conformers with the unfurled E2 and E3 carbonyl O-atoms interfering and repelling each other (Fig. S1), possibly storing mechanical energy from the NOD reaction, and providing spring-like right-handed elastic torque with refurling to assist LeuE11 displacement and E and F helix movements. Both CD loop types are also observed in the dimeric *Ralstonia eutropha* flavoHb-econazole structure (Fig. S2) (42) thus arguing for CD loop conformational changes rather than sequence-specific static structures.

The ferric *E. coli* and *S. cerevisiae* ferric-nitrite flavoHb structures also show differences in the positioning of the proximal LysF7 forming the bridge for electron transfer (ET) (39, 40), suggesting a switching mechanism that is activated by O_2_- and NO-leveraged motions. In the unoccupied ferric structure (Fig. 2*D*, *top*), the LysF7 epsilon ammonium group and GlnE7 amide oxygen atom hydrogen bond a water molecule near the middle of the ∼6-Å-long line-of-flight for ET between the heme carboxylate and FAD C8 methyl group (43) that is open to solvent exchange (38). In this model, the positioning of the electron-rich O atom of water hinders ET. The NO_2_^−^ occupied structure shows a 10° torsional rotation of the F-helix relative to the heme plane (Fig. 2*D*, *bottom*) in which the GlnE7 amide is positioned with TyrB10 hydrogen bonding and stabilizing the Fe^3+^O_2_^−^ intermediate and the LysF7 side chain positioned with the straight line-of-flight for ET passing 0.3 Å beyond the van der Waals' radius of the epsilon ammonium nitrogen, which presumably moves to the position of balance between repulsion by the core and attraction by the annular shell for optimal electron tunneling (44). Electron tunneling paths involving the bridging water molecule and LysF7 have been previously considered (2, 38, 39, 40). The structural differences suggest a LysF7-H_2_O-GlnE7 ET switch mechanism that is activated by the permeation of NO through the G8-E15 gate and by the further upward leveraging LeuE11 with a right-handed torsional twist of the E and F helices that is coupled to CD loop furling.

### Reaction steps and a kinetic model

**Figure 3:**
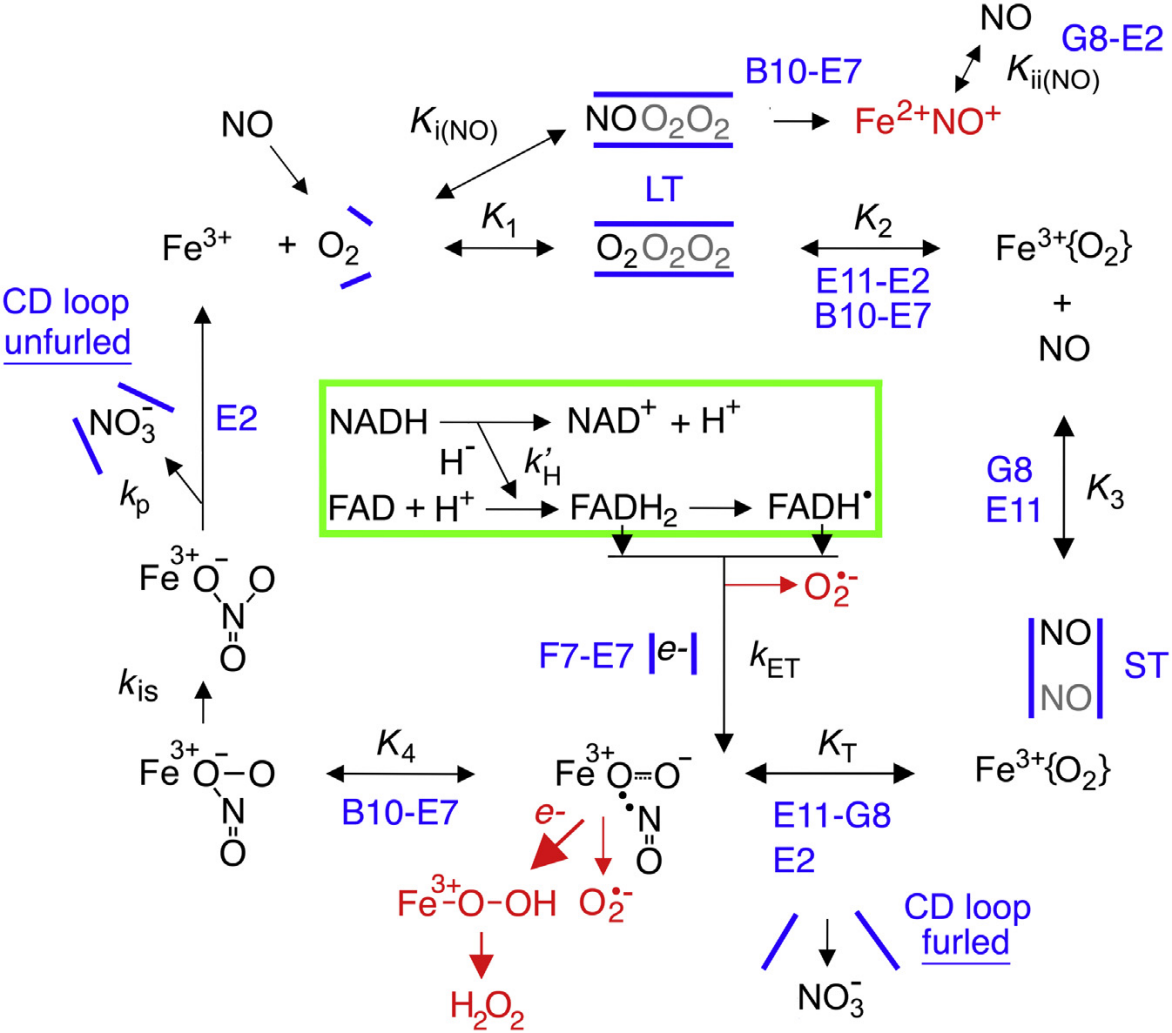
**Reaction steps and kinetic constants in the NOD mechanism model.** The equilibrium constants for the corresponding forward reactions are: *K*_1_, entry of O_2_ into the LT; *K*_2_, O_2_ displacement of LeuE11 and the shifting of the E-helix to widen the G8-E15 entry gate of the ST; *K*_3_, passage of NO through the narrow gate and the allosteric trigger point for structural changes allowing heme reduction (*k*_ET_) and O_2_ ligation with an equilibrium constant of *K*_T_; and *K*_4_, reaction of NO with Fe^3+^O_2_^−^ intermediate to form NO_3_^−^. *k'*_H_, *k*_ET_, *k*_is_, and *k*_p_ are the rate constants for hydride transfer, electron transfer, peroxynitrite isomerization, and NO_3_^−^ dissociation, respectively. *K*_i(NO)_ is the equilibrium constant for NO entering the LT and competitively inhibiting O_2_ migration and the displacement of LeuE11. *K*_ii(NO)_ is the equilibrium constant for NO entry from the CD loop, ST, and other points causing uncompetitive inhibition. *Curly brackets* on O_2_ represent a localization to the distal heme pocket without electron sharing with the heme. *Blue* bars represent the tunnels and gates. The *green* box represents the flavoHb reductase domain.

The structural features of flavoHb described above suggest a catalytic cycle, and testable kinetic model, to explain the cooperative O_2_ and NO activation observed in Figure 1, *A*–*B*. In the kinetic model depicted in Figure 3, nonpolar O_2_ molecules are driven from the aqueous solvent into the LT with an equilibrium constant of *K*_1_, and the leading O_2_ displaces LeuE11 forcing the movement of the E-helix, docks with the ferric iron, the LeuE11 isobutyl group and the TyrB10 hydroxyl, and weakens the G8-E15 gate with an equilibrium constant of *K*_2_. NO then enters and passes through the ST further widening the G8-E15 gate with equilibrium constant *K*_3_ and leveraging the movement of LeuE11, the E-helix, GlnE7, the F-helix, and the LysF7 side chain while triggering furling of the CD loop with the first-order rate constant *k'*_T_ and equilibrium constant (*K*_T_), ET (*k*_ET_), and nitrate release (*k*_p_). Reduction of the ferric heme causes rapid univalent O_2_ reduction forming the Fe^3+^O_2_^−^ intermediate, which then reacts rapidly with the internally sequestered NO with the bimolecular rate constant *k'*_4_ and equilibrium constant *K*_4_ forming the Fe^3+^NO_3_^−^ intermediate that rapidly isomerizes (*k*_is_) (2, 6). Nitrate dissociates from the heme with the first-order rate constant *k*_p_, unfurls the CD loop, and migrates to the putative anion hole. In the proposed reaction scheme, NO inhibits by entering the LT and competing with O_2_ for the ferric heme as expressed by the *K*_i(NO)_ value. The model also allows for excessive NO to permeate a leaky NO entry gate, the nitrate efflux gate, or other pathways and inhibit catalysis with equilibrium constant *K*_ii(NO)_. In the model, inhibitory NO binds ferric heme forming Fe^2+^NO^+^ (45), which readily dissociates (4) and resists reduction to Fe^2+^NO. The observed ET rate depends upon the LysF7-H_2_O-GlnE7 gate positioning and the FAD/FADH_2_ reduction potential, the driving force. The reaction scheme also shows pathways for O_2_^−^ and H_2_O_2_ production as well as the motifs and residues most critical for each step.

We have formulated a steady-state velocity equation for the catalytic cycle with the activating trigger step (*K*_T_) (Equation 1).

(1) $$v=\;V_{\text{max}}\;\left(\text{a}\right)$$

where $\text{a}=\left({\left[\text{NO}\right]}^{2}{\left[{\text{O}}_{2}\right]}^{2}\text{/}K_{1}K_{2}K_{3}K_{\text{T}}K_{4}\right)\text{/}\left(1+(\left[{\text{O}}_{2}\right]{\text{/}K}_{1}\right)+\left({\left[{\text{O}}_{2}\right]}^{2}\text{/}K_{1}K_{2}\right)\;+\left(\left({\left[{\text{O}}_{2}\right]}^{2}\left[\text{NO}\right]){\text{/}K}_{1}K_{2}K_{3}\right)\;+\left(\left({\left[{\text{O}}_{2}\right]}^{2}\left[\text{NO}\right])\text{/}K_{1}K_{2}K_{3}K_{\text{T}}\right)+\left(({\left[{\text{O}}_{2}\right]}^{2}{\left[\text{NO}\right]}^{2}\right){\text{/}K}_{1}K_{2}K_{3}K_{\text{T}}K_{4}\right)\right)$

We have also factored in NO acting as a competitive inhibitor with respect to O_2_ (Equation 2) (46).

(2) $$v=\;V_{\text{max}}\;\left(\text{a}\right)\left(\text{b}\right)$$

where $\text{b}=[\text{NO}]\text{/}\left(K_{3}+\left([\text{NO}]\left(1+\left(\left(K_{2}\text{/}[{\text{O}}_{2}]\right)\left(1+\left([\text{NO}]\text{/}K_{\text{i}(\text{NO})}\right)\right)\right)\right)\right)\right)$

And, we have allowed NO and O_2_ to each act as uncompetitive inhibitors of catalysis (Equations 3 and 4, respectively).

(3) $$v\;=\;V_{\text{max}}\;\left(\text{a}\right)\left(\text{b}\right)\left(\text{c}\right)$$

where $\text{c }=\;1\text{/}\left(1+\left(\left[\text{NO}\right]{\text{/}K}_{\text{ii}\left(\text{NO}\right)}\right)\right)$

(4) $$v\;=\;V_{\text{max}}\;\left(\text{a}\right)\left(\text{b}\right)\left(\text{c}\right)\left(\text{d}\right)$$

where $\text{d }=\;1\text{/}\left(1+\left(\left[{\text{O}}_{2}\right]\text{/}K_{\text{i}\left(\text{O}2\right)}\right)\right)$

These additional independent statistical factors and the equilibrium constants *K*_ii(NO)_ and *K*_i(O2)_ have been included for situations in which NO and O_2_ do not compete for LT or ST entry, or iron binding, but nevertheless inhibit catalysis. In addition, we have introduced a factor and equilibrium constant *K*_a(O2)_ for rare situations in which O_2_ causes an additional NO-independent activation of catalysis possibly by occupying unique binding sites or cavities (Equation 5). Equations (3), (4), (5) assume a normal hyperbolic binding and inhibition or activation response.

(5) $$v\;=\;V_{\text{max}}\;\left(\text{a}\right)\left(\text{b}\right)\left(\text{c}\right)\left(\text{e}\right)$$

where $\text{e}=\;\text{1}\;+\;\left(\left[{\text{O}}_{2}\right]\text{/}K_{\text{a}\left(\text{O}2\right)}\right)$

### Fit of the mechanistic kinetic model with steady-state kinetic data

The kinetic data for the WT flavoHb in Figure 1, *A*–*B* are transformed to the respective log–log plots in Figure 1, *C*–*D*. Logarithmic plots were preferred since they display large ranges of substrate concentrations and velocities with a much greater resolution (46) and allow for same-scale visual comparisons. We have estimated the kinetic constants in Table 1 by fitting the velocity equation to the steady-state kinetic data with the resulting fits shown as *dashed* lines in Figure 1, *C*–*D*. For fitting, we assumed *K*_1_ << *K*_2_. *K*_1_ was taken to be 2 μM, the value estimated from O_2_ association kinetics where *V*_max_/*k'*_O2_ was the theoretical *K*_m_(O_2_) of ∼2 μM at 20 °C (4). It should be noted that the theoretical *v* is most affected by the *K*_1_ value at low O_2_ and NO concentrations. When *K*_1_ is assigned a 10-fold larger value and *K*_2_ = 120 μM, *v* is <2-fold smaller with the largest effects at low [O_2_] and [NO]. And, smaller *K*_1_ values relative to a large *K*_2_ diminish any effects of *K*_1_ on *v*. A range of values for *K*_3_ and *K*_4_ were required to model the data at 20 °C as shown by the *black* and *red dashed* lines in Figure 1*D* and provided in the data in Table 1. A loss of harmonics and frustration in enzyme motion (47) may explain the deviations from a more ideal kinetic behavior at the subphysiological 20 °C. A *k'*_NOD_ value of 1 to 2 × 10^10^ M^−1^ s^−1^ can be estimated from the *V*_max_/*K*_4_ ratio at 37 °C, a rate constant approaching the value of 6.9 × 10^10^ M^−1^ s^−1^ for the bimolecular reaction of NO and free O_2_^−^ (48).

**Table 1:** Kinetic constants for WT, and G8 and E11 variant flavoHb-NODs

Constant	WT	LeuG8	AlaG8	TrpE11	AlaE11
*V*_max_^a^	580 (160)	650 (350)	550 (85–90)	500 (280)	300 (10–11)
*K*_1_^b^	2 (2)	2 (2)	2 (2)	2 (2)	2 (2)
*K*_2_^b^	120 (20)	200 (50)	80 (30)	400 (225)	100 (10)
*K*_3_^b^	0.25 (0.1–0.4)	0.9 (1.0)	0.1–0.2 (0.12–0.7)	12 (12)	0.04–0.15 (0.04–0.5)
*K*_T_	0.1 (0.1)	0.001 (0.01)	0.2 (0.2)	0.0001 (0.0001)	0.5 (1.5)
*K*_4_^b^	0.03–0.06 (0.06–0.3)	0.01–0.02 (0.03–0.07)	0.03–0.12 (0.03–0.10)	0.02–0.2 (0.01–0.25)	0.01–0.04 (0.01–0.03)
*K*_i(NO)_^b^	0.7–1.2 (0.3–1.0)	0.4–20 (0.1–1.0)	0.15–1.0 (0.1–0.25)	0.2–3 (0.2–0.5)	0.4–10 (1–2)
*K*_ii(NO)_^b^	10 (10)	100 (100)	5 (10)	100 (3)	100 (3)
*K*_i(O2)_^b^	-	-	-	-	300
	-	-	-	-	-
*K*_a(O2)_^b^	-	-	-	-	-
	-	-	-	-	(1000)
*k'*_NOD app_^c^	2.3 (0.4–1.6)	0.72 (0.35)	2.3–5.5 (0.12–0.75)	0.042 (0.023)	2.0–7.5 (0.02–0.28)
*k'*_NOD_^d^	9.7–19 (0.5–2.7)	33–65 (5–12)	4.6–18 (0.9–3)	2.5–25 (1.1–28)	7.5–30 (0.3–1.1)

NOD, NO dioxygenase.

Values in parentheses are for 20 °C.

a NO heme^−1^ s^−1^.

b Micromolar.

c Calculated from *V*_max_/*K*_3_ and expressed in units of 10^9^ M^−1^ s^−1^.

d Calculated from *V*_max_/*K*_4_ and expressed in units of 10^9^ M^−1^ s^−1^.

**Figure 4:**
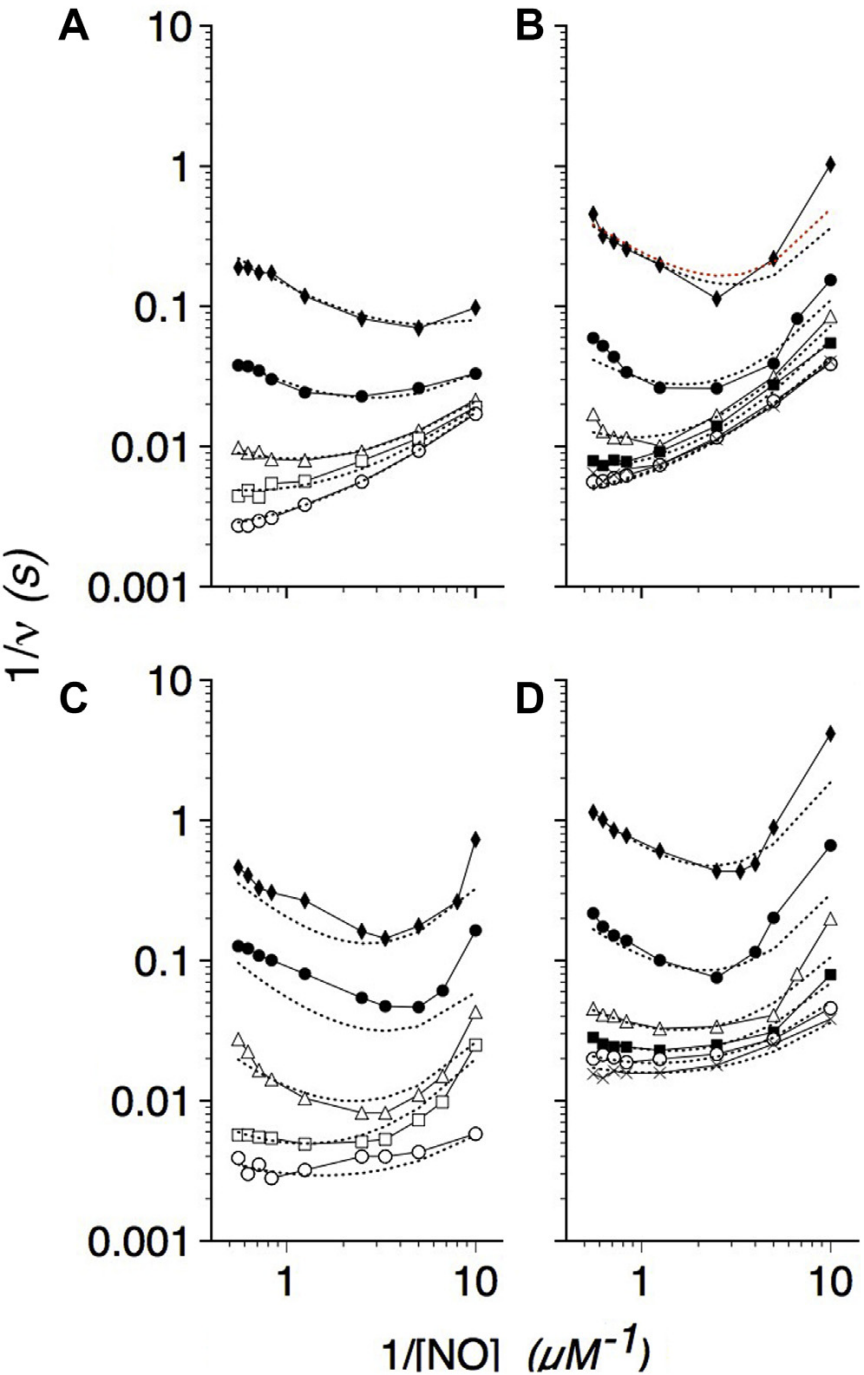
**O**_**2**_**and NO dependence of G8 mutant NOD activities.** Kinetic model calculations fitted to Lineweaver–Burk log–log plots of LeuG8 (*A* and *B*) and AlaG8 (*C* and *D*) NOD activities *versus* [NO] at 37 (*A* and *C*) and 20 °C (*B* and *D*). O_2_ concentrations were as described in the legend to Figure 1. Kinetic constants determined from the data and model are given in Table 1.

**Figure 5:**
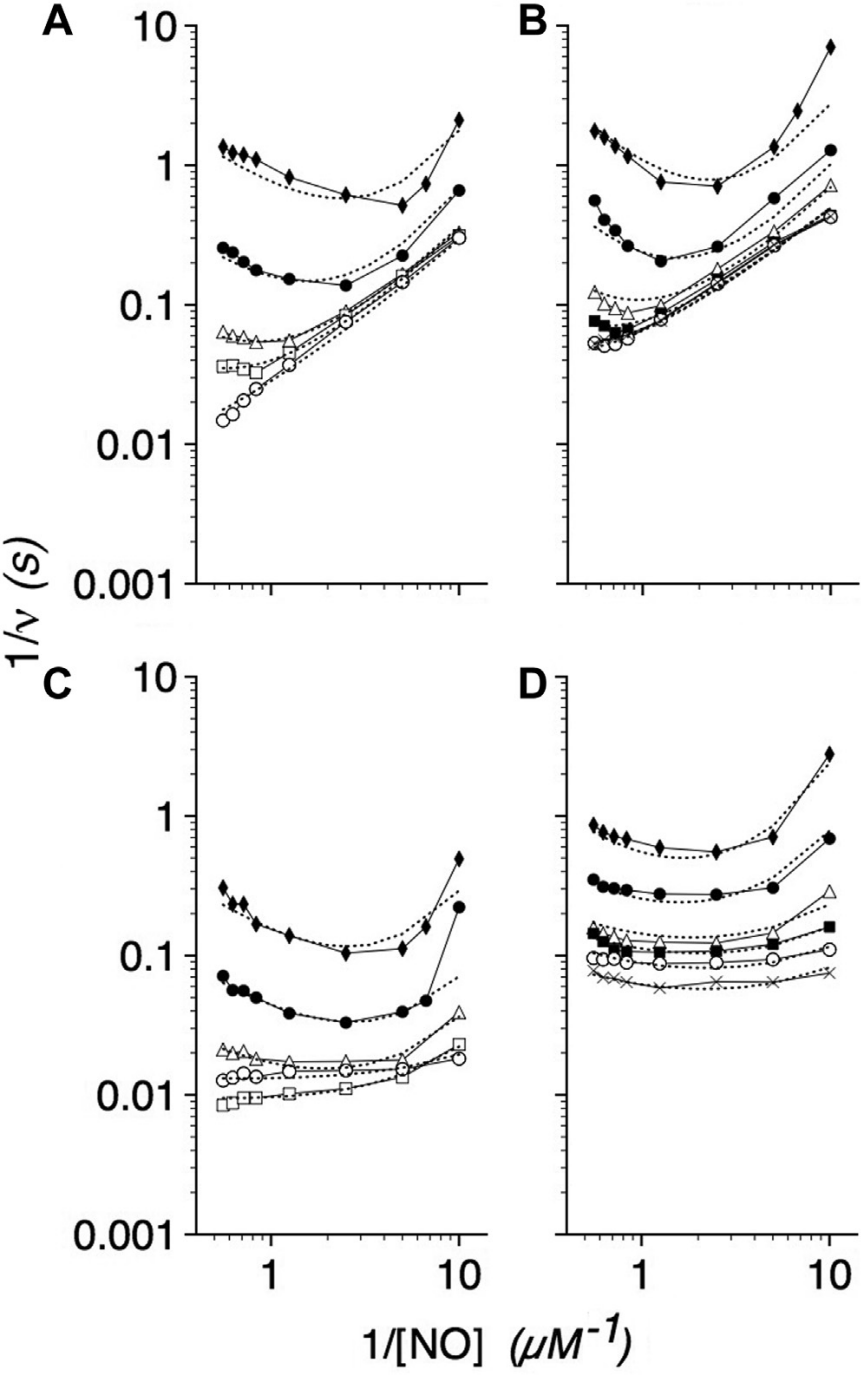
**O**_**2**_**and NO dependence of E11 mutant NOD activities.** Kinetic model calculations fitted to Lineweaver–Burk log–log plots of TrpE11 (*A* and *B*) and AlaE11 (*C* and *D*) NOD activities *versus* [NO] at 37 (*A* and *C*) and 20 °C (*B* and *D*). O_2_ concentrations were as described in the legend to Figure. 1. Kinetic constants determined from the data and model are given in Table 1.

Similar steady-state kinetic data for G8 and E11 substitution mutants are shown in Figures 4 and 5, respectively, and kinetic constants used to fit the data are also given in Table 1. Substituting ValG8 with the larger Leu residue increases *K*_3_, the apparent *K*_m_(NO), whereas substituting ValG8 with a smaller Ala decreases *K*_3_ as expected for a role for ValG8 in gating NO entry. The critical function of ValG8 in the allosteric mechanism is also suggested by the large changes in the *K*_T_ values in the mutants, and the apparently large frustration of AlaG8 at 20 °C. The data also reveal a role for ValG8 in determining the susceptibility to NO inhibition. AlaG8 shows smaller *K*_i(NO)_ and *K*_ii(NO)_ values, whereas the bulkier LeuG8 shows larger values. The effect is more discernible at 37 than 20 °C, again suggesting greater frustration at the lower temperature. A greater resistance of LeuG8 to NO inhibition also confers a higher *V*_max_ at both temperatures; however, both the NO sensitivity, as indicated by small *K*_i(NO)_ and *K*_ii(NO)_ values, and the impaired allosteric activation, as indicated by large *K*_T_ values, lower the *V*_max_ for AlaG8. The data support the roles for G8 shown in the kinetic model in Figure 3 and structure model in Figure 2*C*. G8 substitutions also cause relatively smaller effects on the O_2_ dependence (*K*_2_) suggesting effects on E-helix and LeuE11 movement.

Substituting LeuE11 with the larger Trp caused large increases in *K*_3_ and *K*_2_ and a large decrease in *K*_T_ (Table 1). Consistent with the structure-based kinetic model (Fig. 2*C* and Fig. 3), TrpE11 hinders NO access, increases the O_2_ requirement for activity, but nevertheless favors the activation by O_2_ and NO. In contrast, the smaller Ala substitution shows the opposite effects. Larger *K*_2_ values for the TrpE11 mutant indicate larger O_2_ requirements for displacement and E-helix movement. The small *K*_T_ suggests a strong, and effective, response to NO movements. The data support roles for LeuE11 as the O_2_ and NO activated trigger for the allosteric mechanism and as a part of the NO access tunnel. However, the data for the AlaE11 variant were unusual in that the kinetic model could only be fitted to the data by allowing for O_2_ inhibition at 37 °C and O_2_ activation at 20 °C.

**Figure 6:**
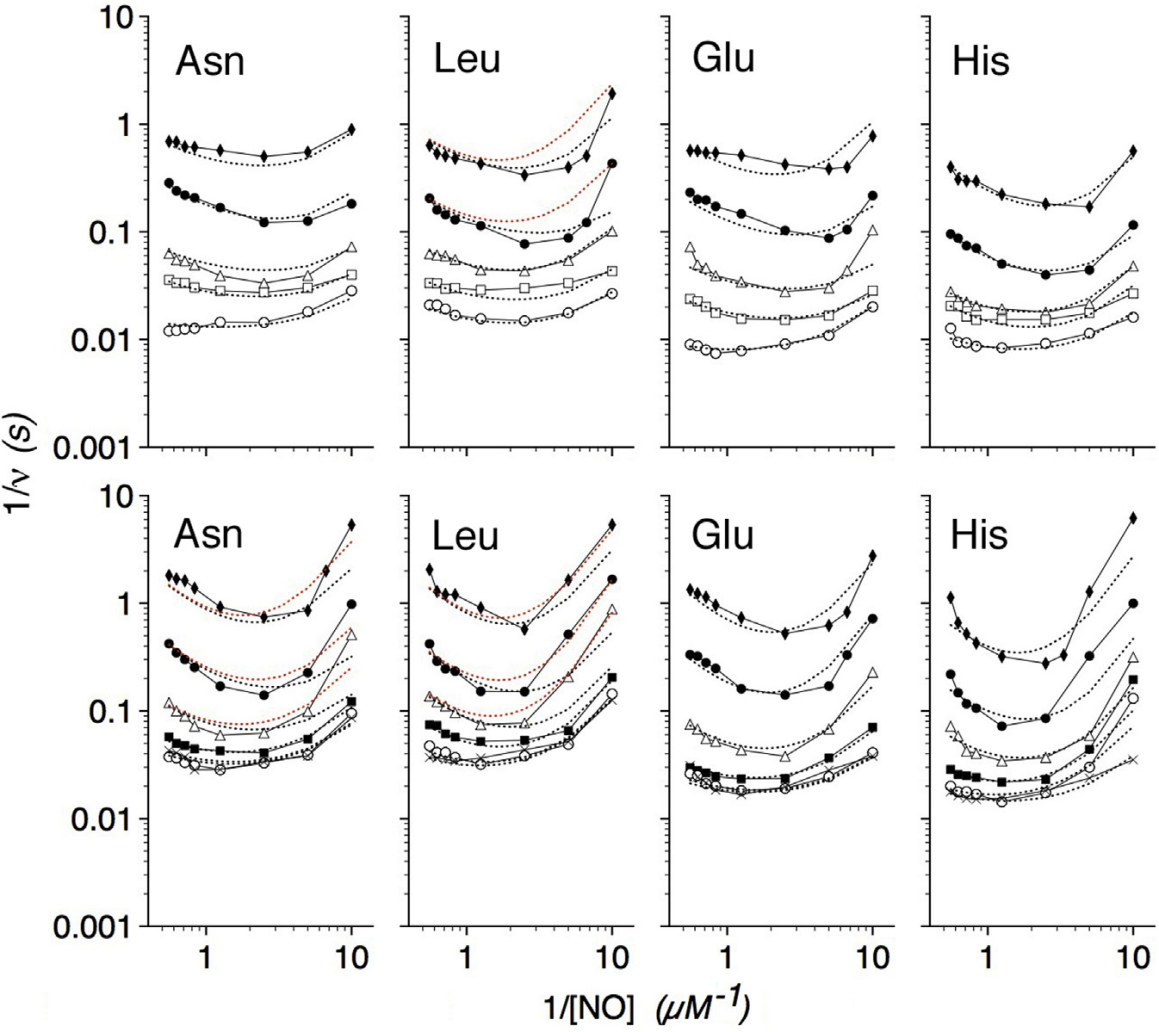
**O**_**2**_**and NO dependence of E2 mutant NOD activities.** Kinetic model calculations fitted to Lineweaver–Burk log–log plots of E2 substitution NOD activities *versus* [NO] at 37 (*top panels*) and 20 °C (*bottom panels*). O_2_ concentrations were as described in the legend to Figure 1. Kinetic constants determined from the data and model fitting are given in Table 2.

GlnE2 within the CD loop was tested for its proposed role in the allosteric mechanism. Data for E2 substitution flavoHbs are shown in Figure 6, and kinetic constants used to fit the data are given in Table 2. Substitution of the highly conserved GlnE2 with Asn, Leu, His, and Glu causes larger *K*_T_ values representing an impaired activation of ET by NO and O_2_, thus producing a two- to threefold decrease in the *V*_max_ values and proportionally smaller *K*_3_ and *K*_4_ values. *K*_2_ values increase two- to threefold except for the His substitution at 37 °C. The data demonstrate an important role for GlnE2 in the NOD mechanism. The larger *V*_max_ values and lesser effects of oppositely charged His and Glu substitutions on *K*_T_ suggest that Gln allows optimal furling–unfurling of the CD loop rather than interacting directly with nitrate. Furthermore, the consistently smaller *K*_ii(NO)_ values of the E2 variants suggest a CD loop structure dysfunction that opens a migration pathway for NO.

**Table 2:** Kinetic constants for E2 flavoHb-NOD variants

Constant	AsnE2	LeuE2	HisE2	GluE2
*V*_max_^a^	135 (50)	155 (60)	225 (115)	250 (90)
*K*_1_^b^	2 (2)	2 (2)	2 (2)	2 (2)
*K*_2_^b^	300 (70)	275 (60)	120 (70)	350 (70)
*K*_3_^b^	0.015–0.07 (0.05–0.08)	0.05–0.08 (0.15–0.2)	0.10–0.15 (0.13–0.5)	0.05–0.15 (0.07–0.3)
*K*_T_	2.0 (2.0)	1.5 (2.0)	0.7 (0.7)	0.7 (1.0)
*K*_4_^b^	0.01 (0.01–0.05)	0.01–0.03 (0.03)	0.03–0.04 (0.03–0.05)	0.02–0.03 (0.03–0.07)
*K*_i(NO)_^b^	1–10 (0.5–2)	3–10 (0.35–0.6)	0.7–2 (0.5–2)	0.8–5 (0.3–2)
*K*_ii(NO)_^b^	10 (3)	2 (3)	3 (3)	10 (3)
*k'*_NOD app_^c^	1.9–9 (0.63–1.0)	1.9–3.1 (0.3–0.4)	1.5–2.3 (0.45–1.7)	1.7–5 (0.3–1.3)
*k'*_NOD_^d^	13.5 (1.0–5.0)	5.2–15.5 (2.0)	5.6–7.5 (2.3–3.8)	8.3–12.5 (1.3–3.0)

NOD, NO dioxygenase.

Values in parentheses are for 20 °C.

a NO heme^−1^ s^−1^.

b Micromolar.

c Calculated from *V*_max_/*K*_3_ and expressed in units of 10^9^ M^−1^ s^−1^.

d Calculated from *V*_max_/*K*_4_ and expressed in units of 10^9^ M^−1^ s^−1^.

**Figure 7:**
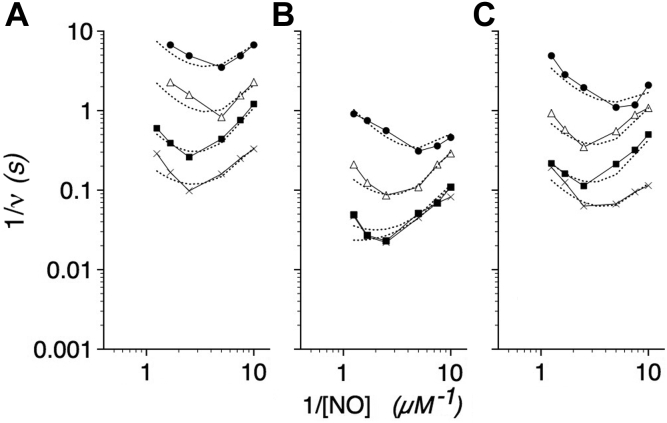
**O**_**2**_**and NO dependence of B10 and E7 mutant NOD activities.** Kinetic model calculations fitted to log–log Lineweaver–Burk plots of PheB10 (*A*), LeuE7 (*B*), and HisE7 (*C*) NOD activities *versus* [NO] at 20 °C. O_2_ concentrations were as described in the legend to Figure 1. Kinetic constants determined from the data and model fitting are given in Table 3.

TyrB10 and GlnE7 are important for stabilization of the Fe^3+^O_2_^−^ intermediate in (flavo)Hbs (1, 4, 49). In the proposed kinetic model (Fig. 3), mutants with decreased Fe^3+^O_2_^−^ stability and larger O_2_ dissociation rate constants are expected to show a higher O_2_ dependence and larger *K*_2_ values, a greater competitiveness of NO for Fe^3+^, and potentially Fe^2+^, and smaller *K*_i(NO)_ and *K*_ii(NO)_ values, and an inefficiency of NO dioxygenation and larger *K*_4_ values. In addition, GlnE7 mutants are expected to show impaired ET switching and altered *k*_ET_ and *K*_T_ values. Data for B10 and E7 flavoHb variants measured at 20 °C are shown in Figure 7, and kinetic constants used to fit the data are given in Table 3. Indeed, the mutant NODs are highly susceptible to NO inhibition and show small *K*_i(NO)_ and *K*_ii(NO)_ values, which also limited the O_2_ and NO concentrations as well as the temperature that could be tested. PheB10 shows a 4-fold larger *K*_2_ value while LeuE7 and HisE7 show more modest two- to threefold increases. HisE7 shows a larger *K*_T_ value possibly reflecting impaired ET while LeuE7 shows negligible effect on the *K*_T_ value suggesting normal switching at 20 °C, but not 37 °C. *K*_3_ values are unexpectedly increased in the PheB10 and E7 mutant NODs and may reflect a role for TyrB10 and GlnE7 in facilitating O_2_ binding and the displacement of LeuE11 from the ferric iron. Of interest, the calculated *V*_max_ values are not decreased in the mutants indicating the capacity of elevated O_2_ concentrations to overcome any defects in O_2_ binding and Fe^3+^O_2_^−^ stabilization. The greater susceptibility to NO inhibition at a higher [NO], a lower [O_2_], and 37 °C may be explained by Fe^2+^NO formation.

**Table 3:** Kinetic constants for the B10 and E7 flavoHb-NOD variants

Constant	PheB10	LeuE7	HisE7
*V*_max_^a^	180	250	200
*K*_1_^b^	2	2	2
*K*_2_^b^	400	45	60
*K*_3_^b^	1.0–1.5	0.7–1.3	0.3–0.9
*K*_T_	0.1	0.1	0.5
*K*_4_^b^	0.10–0.12	0.1	0.10–0.12
*K*_i(NO)_^b^	0.06–0.12	0.05–0.6	0.006–0.017
*K*_ii(NO)_^b^	0.2	0.5	0.5
*k'*_NOD app_^c^	0.12–0.18	0.19–0.35	0.22–0.67
*k'*_NOD_^d^	1.5–1.8	2.5	1.7–2.0

NOD, NO dioxygenase.

All values are for 20 °C.

a NO heme^−1^ s^−1^.

b Micromolar.

c Calculated from *V*_max_/*K*_3_ and expressed in units of 10^9^ M^−1^ s^−1^.

d Calculated from *V*_max_/*K*_4_ and expressed in units of 10^9^ M^−1^ s^−1^.

**Figure 8:**
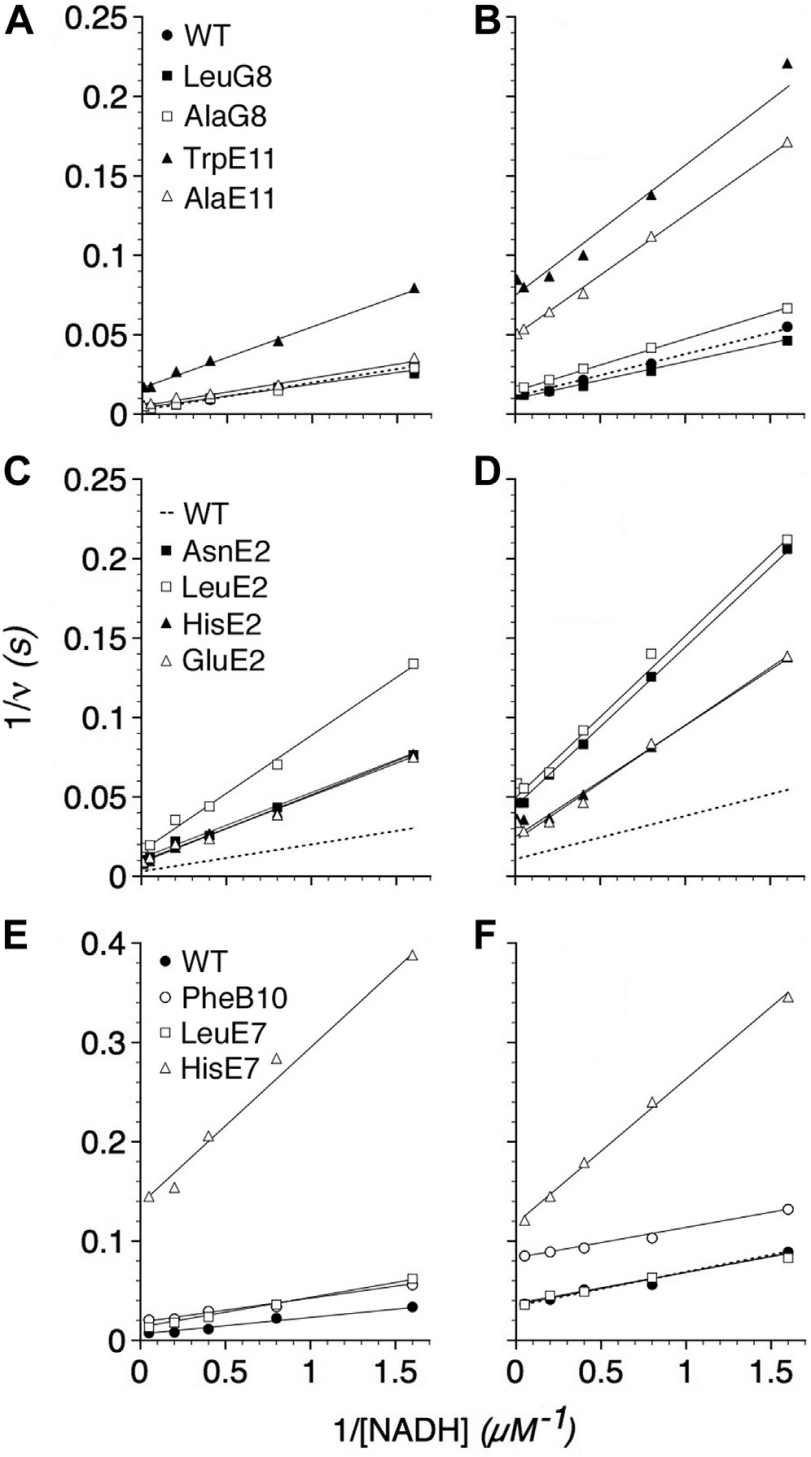
**Effects of G8, E11, E2, B10, and E7 substitutions on the NADH dependence of the NOD activities.** Lineweaver–Burk plots of NOD activities with varying NADH at 37 (*A*, *C*, and *E*) and 20 °C (*B*, *D*, and *F*). *Dashed lines* (*C*–*D*) represent WT data (*A*–*B*).

In the kinetic model (Fig. 3), the velocity (*v*) also serves as a measure of the rate of ET as expressed as a first-order time-averaged rate constant for ET, and the minimal *k*_ET_ value (4, 5). Furthermore, changes in the NADH dependence of the velocities of variant NODs reveal alterations in the ET gating efficiency, where the *v* approximates the *k*_ET_ value owing to the gating mechanism, provided that hydride transfer, and the *k'*_H_ value, remains constant for FAD reduction (Fig. 3). Thus, [NADH]*k'*_H_ determines the steady-state FAD/FADH_2_ ratio, and the reduction potential that directly influences the ET rate through a tunneling matrix (44), and the absolute *k*_ET_ value. In the steady state, Δ*v*/Δ[NADH] values thus provide a sensitive, albeit indirect, measure of the putative LysF7-H_2_O-GlnE7 ET gate function during catalysis (Fig. 2*D*) where a smaller Δ*v*/Δ[NADH] value indicates a decreased rate of ET, an inefficient tunneling matrix, and dysfunctional gating mechanism. As shown in Figure 8, the TrpE11 mutant, all E2 substitutions, and the HisE7 mutant show increased slopes in 1/*v versus* 1/[NADH] plots at 37 and 20 °C and smaller Δ*v*/Δ[NADH] values (Table 4). In addition, the AlaE11 substitution shows an increased slope and smaller Δ*v*/Δ[NADH] value at 20 °C. Dysfunction of ET in the TrpE11, LeuE2, LeuE7, and HisE7 substitutions is observed at 37 °C, and even greater effects of all E11 and E2 substitutions and the HisE7 substitution are observed at 20 °C. LeuE7 shows a small stimulatory effect at 20 °C compared with GlnE7. The G8 substitutions show only minor effects, and those are observed at 20 °C with Ala decreasing and Leu increasing the efficiency. The PheB10 substitution also shows only minor effects.

**Table 4:** NADH dependence of WT and mutant NODs

NOD	Δv/Δ[NADH] (s^-1^ μM^-1^)
WT	59 (36)
LeuG8	60 (44)
AlaG8	60 (31)
TrpE11	26 (12)
AlaE11	53 (13)
AsnE2	24 (10)
LeuE2	14 (10)
GluE2	24 (14)
HisE2	23 (14)
WT^a^	59 (30)
PheB10^a^	44 (33)
LeuE7^a^	32 (33)
HisE7^a^	6.4 (7.1)

NOD, NO dioxygenase.

a Values are for 130 nM NO. Values in parentheses are for 20 °C.

The results demonstrate roles for GlnE7, LeuE11, and GlnE2 in ET consistent with the proposed mechanism and kinetic model. The analysis also indicates that higher NADH concentrations, and thus more negative flavin redox potentials, can drive catalysis and ET in a defective gate mechanism. The low-temperature dependence of Δ*v*/Δ[NADH] also indicates a relatively low energy barrier for the hydride and electron transfer steps.

### Kinetics of hydride and ET

**Figure 9:**
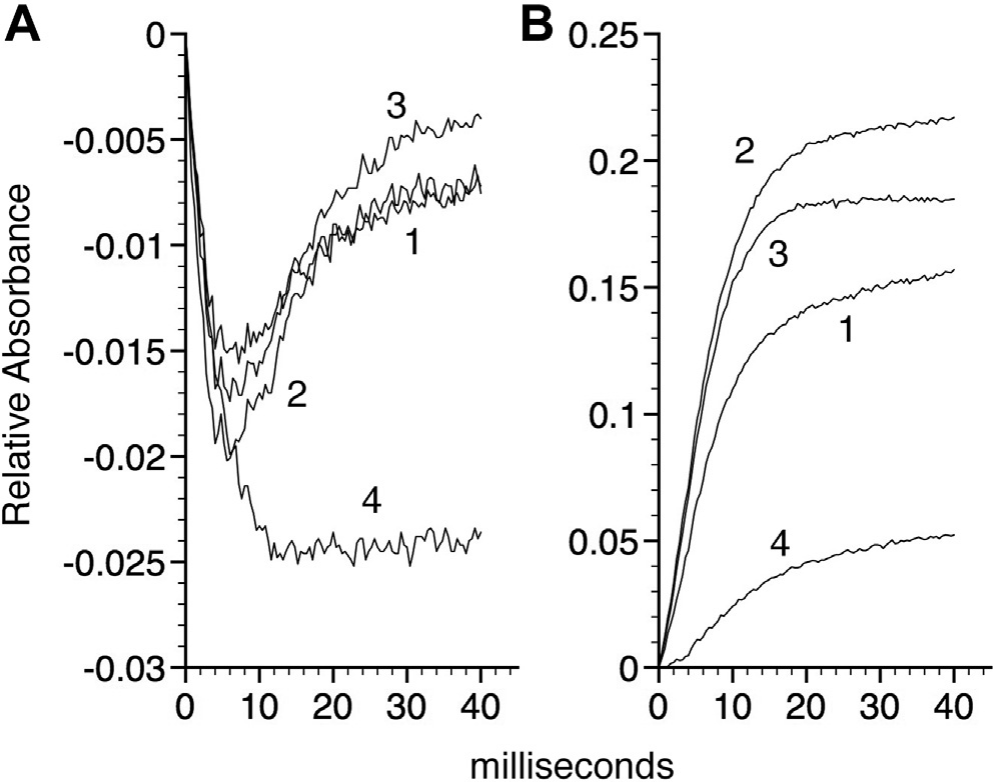
**Kinetics of the reduction steps in WT, and B10 and E7 substitution mutants.***A*, FAD reduction and oxidation as measured by the absorbance changes at 460 nm and *B*, heme reduction as detected by the increase in absorbance at 430 nm for the WT (*line 1*), PheB10 (*line 2*), LeuE7 (*line 3*), and HisE7 (*line 4*) flavoHbs.

As previously reported (4), NADH reduces FAD to FADH_2_ with a large bimolecular rate constant for hydride transfer (*k′*_H_) of 1.5 × 10^7^ M^−1^ s^−1^ as detected by the transient decrease in FAD absorbance at 460 nm (Fig. 9*A*, *line 1*). Similar FAD reduction rates are observed for the PheB10, LeuE7, and HisE7 variants (*lines 2–4*, respectively) with the HisE7 mutant showing a greater extent of FAD reduction. FADH_2_ rapidly reduces the WT flavoHb ferric heme to the ferrous state with a maximum *k*_ET_ value of 120 s^−1^ at 20 °C as measured by the transient increase in absorbance at 430 nm (Fig. 9*B*, *line 1*), similar to the 136 s^−1^ value previously reported (4). In comparison, the apparent *k*_ET_ values for the PheB10, LeuE7 and HisE7 variants are 160, 140 and a biphasic 1.7 s^−1^ or 50 s^−1^, respectively (*lines 2–4*). It is noteworthy that the HisE7 variant showed a biphasic behavior with an initial slow reduction followed by a more rapid phase with incomplete reduction (*line 4*) despite achieving a more negative FAD/FADH_2_ reduction potential (Fig. 9*A*, *line 4*). The HisE7 variant appears to favor the *off* conformation (see Discussion). In contrast, WT, LeuE7, and PheB10 flavoHbs appear to favor an *on* conformation in the resting state. Both the *k′*_H_ and *k*_ET_ values of the WT, LeuE7, and PheB10 flavoHbs are within the range of values required to support the NOD turnover rates measured at 20 °C (Tables 1 and 3). The transient kinetic data further support a role for GlnE7 movements in the ET gate function. ET rates, and *k*_ET_ values, depend upon the resting-state structure and the FAD/FADH_2_ potential.

### O_2_, NO, and CO binding

The kinetic model predicts specificity and dynamic changes in ligand migration pathways during catalysis. Indeed, a heterogeneity in ligand migration pathways was evident in previous biphasic ligand binding kinetics (4, 5). However, biphasic kinetics was attributed to a structural heterogeneity in the isolated flavoHbs, rather than to the existence of multiple and dynamic pathways, as recently argued for CO migration in Cygb (50).

A critical reanalysis of the ligand binding kinetics of flavoHb shows evidence of dynamic changes and multiple pathways. Following the high-energy laser photolysis perturbation, flavoHb rebinds O_2_, NO, and CO with biphasic kinetics (Fig. S3) with transition times (τ_T_) for the fast and slow phases in the 10- to 200-μs range (Table 5). For O_2_ rebinding to the WT flavoHb, the τ_T_ is ∼55 μs for both high and low [O_2_] conditions, while the fraction of flavoHbFe^3+^O_2_^−^ formed in the fast phase decreases by ∼50% with lower [O_2_] (Fig. S4). These data demonstrate a relatively slow, but large, loss in pathways available for O_2_ migration and rebinding following the photolytic perturbation. Moreover, the slow phase of O_2_ (1 mM) rebinding is 86% inhibited by 100 μM CO, whereas the fast phase is only inhibited by ∼10%, (Table 5, Tables 5 and S5) indicating that the relevant migration pathway(s) for the NO dioxygenation mechanism are represented by the slow phase since CO competitively and potently inhibits the NOD activity with respect to O_2_ (5), and presumably inhibits O_2_ migration through the LT as well as O_2_ binding to the iron. The PheB10 and HisE7 variants show similar *k'*_slow_ (O_2_) values, but show only ∼50% inhibition of O_2_ migration and binding by CO in the slow phase suggesting availability of pathways for O_2_ migration, such as the ST, CD loop, or E-helix heme interface, in addition to the LT. The mutations, iron redox state, ligand types, and concentration affect the τ_T_ for ligand binding and the photolysis fraction (Φ_dis_) in ways that are not readily interpretable. We suppose that the large τ_T_ values of >8 μs are due to gases transiently maintaining nonspecific pathways for gas migration initially opened by the quake of photolysis (see Discussion).

**Table 5:** Kinetic constants for ligand binding to flavoHb-NODs

Ligand pair	Φ_dis_	*k′*_fast_	τ_T_	*k′*_slow_	*k*	*K*_d_
	%	μM^−1^ s^−1^	μs	μM^−1^ s^−1^	s^−1^	nM
Fe^2+^ O_2_						
WT	1.6	33 (90%)	70 ± 5	14 (10%)	0.20	14
PheB10	4.9	180 (79%)	20 ± 10	13 (21%)	3.5^a^	269
LeuE7	3.0	28 (85%)	100 ± 20	16 (15%)	1.4	88
HisE7	0.9	75 (60%)	10 ± 5	12 (40%)	5.8	483
Fe^2+^ NO						
WT	4.4	31 (57%)	40 ± 10	8.5 (43%)	0.0002 (4)	0.024
PheB10	2.4	145 (78%)	8	25 (22%)	0.0001 (4)	0.004
LeuE7	2.4	191 (59%)	8	39 (41%)	n.d.	
HisE7	2.0	45 (53%)	8 ± 3	12 (47%)	n.d.	
Fe^3+^ NO						
WT	22	46 (57%)	18 ± 3	12 (43%)	∼4000 (4)	3.3 × 10^5^
PheB10	21	64 (58%)	18 ± 3	13 (42%)	n.d.	
LeuE7	16	72 (63%)	13 ± 3	13 (37%)	n.d.	
HisE7	16	70 (61%)	13 ± 8	7.5 (39%)	n.d.	
Fe^2+^ CO						
WT	13	14 (72%)	175 ± 25	1.4 (28%)	0.055 (32%)	12–39
					0.017 (65%)	
PheB10	15	67 (65%)	75 ± 25	3.0 (35%)	0.029 (29%)	1.3–9.7
					0.004 (71%)	
LeuE7	11	5.8 (77%)	150 ± 50	1.0 (23%)	0.076 (13%)	16–76
					0.016 (87%)	
HisE7	15	15 (44%)	10 ± 5	1.2 (56%)	0.058 (35%)	1.0–48
					0.015 (65%)	

NOD, NO dioxygenase.

a Smaller than previously reported (4) because the previous value (34 s^−1^) contained an error owing to CO binding to the deoxy form. *n.d.* indicates values not determined.

Slow phases of ligand binding were accordingly used for the revised calculations of *K*_d_ values and for analyzing the enzyme mechanism. PheB10, LeuE7, and HisE7 substitutions show similar *k′*_slow_ (O_2_) values, but show larger *k*(O_2_) values, and respective 19-, 6-, and 35-fold larger *K*_d_(O_2_) values than the WT enzyme (Table 5). The results further support roles for a TyrB10 and GlnE7 hydrogen bond network in stabilizing the Fe^3+^O_2_^−^ intermediate (1, 49), and together with the kinetic model in Figure 3, help to explain the steady-state kinetic data in Figure 7 and Table 3. Moreover, the 29-fold larger *k*(O_2_) value measured for the HisE7 variant relative to that for GlnE7 (Table 5) further suggests that the HisE7 variant, in its resting state, favors the *off* conformation with positively charged HisE7 in proximity to the negatively charged heme propionate as modeled for GlnE7 in Figure 2*D* (*top*). In addition, the comparable *k′*_slow_ (NO) and *k′*_slow_ (O_2_) values and the large *k*(NO) values for the ferric flavoHbs (Table 5) may account, in part, for the reversible NO inhibition reported in Figure 1 and modeled in Figure 3. Conversely, comparable *k′*_slow_ values for O_2_ and NO and extremely small *k*(NO) and *K*_d_(NO) values for the ferrous flavoHb (Table 5), and an absence of irreversible NO inhibition, largely preclude the previously suggested ping-pong mechanism (3, 4). Applying the *k*(NO) values, we can calculate 90% activity recovery following NO binding in 3.2 h for the ferrous NO species and a mere 0.6 ms for the ferric NO form. With NO elimination *via* reduction (≤0.1 s^−1^) (4, 5), 90% reversal in the steady state would require 23 s. However, NO inhibition is competitive with O_2_ and seamlessly transient under the steady-state conditions of Figures 1 and Figure 4, Figure 5, Figure 6, Figure 7 indicating a predominant competition for the LT entry and ferric iron as modeled in Figure 3. NO and O_2_ show remarkably similar *k′*_slow_ values for Fe^3+^ and Fe^2+^, respectively, suggesting a similar entry and migration through the LT and interactions with LeuE11.

### Role of LysF7 in ET control

**Figure 10:**
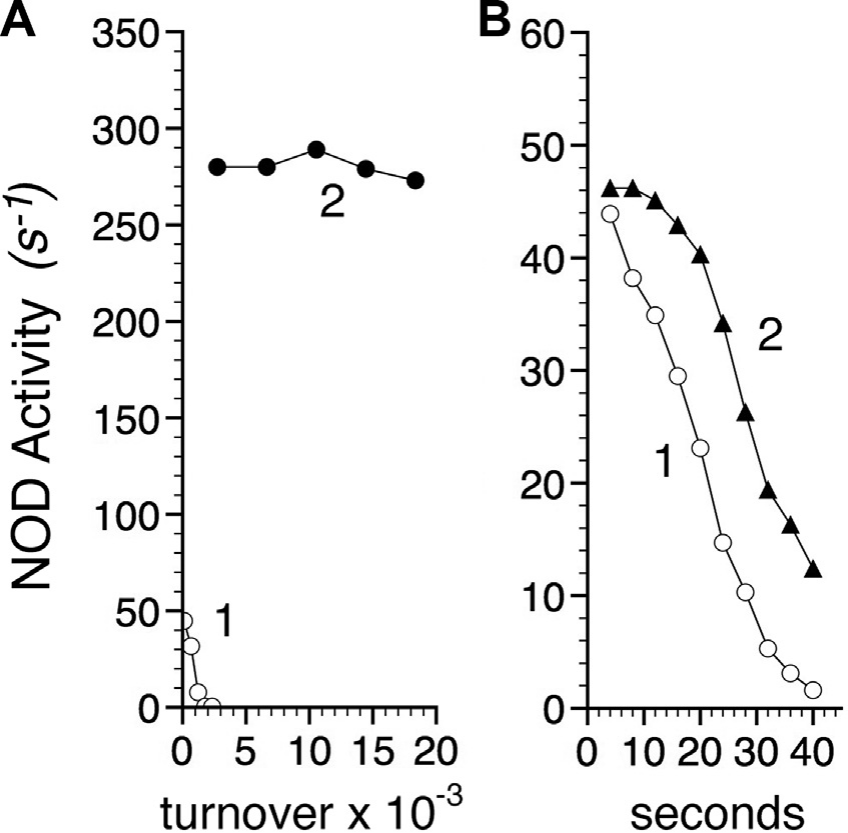
**Autocatalytic inactivation of an AsnF7 substitution mutant NOD.***A*, NOD activities of AsnF7 (*line 1*) and LysF7 (*line 2*) flavoHbs were followed at 37 °C with 200 μM O_2_ with between 1 and 2 μM NO, and *B*, activity was measured at various times in the absence (*line 1*) or presence of 1000 U of catalase (*line 2*).

LysF7 was investigated for its role in gating ET with an AsnF7 substitution that removes the proposed toggling epsilon ammonium group in the gate structure (Fig. 2*D*). The starting activity of the AsnF7 enzyme is 6-fold less than the LysF7 (WT) enzyme and rapidly inactivated. Ninety percent of the starting activity is lost in less than 1000 turnovers or ∼30 s (Fig. 10*A*, compare *line 1* with *line 2*). In the absence of NO, the AsnF7 enzyme loses 85% of its NOD activity in 90 s with apparent zero order kinetics (Fig. 10*B*, *line 1*). Thus, NO is not essential for the inactivation, but NO greatly increases the inactivation. Catalase provides modest protection (Fig. 10*B*, compare *lines 1* and *2*) suggesting formation of Fe^3+^-OOH, and H_2_O_2_, and oxidative damage to the heme. Indeed, flavoHb-NOD activity is sensitive to H_2_O_2_, and the heme ligand miconazole (51) and the peroxidase substrate ascorbate both protect in the resting state (Fig. S5). The AsnF7 variant shows other indications of changes in ET fidelity or control including a 2.8-fold greater NADH oxidase activity (0.76 *versus* 0.27 s^−1^), a 2.4-fold greater NO reductase activity (0.45 *versus* 0.19 s^−1^), a fourfold higher methyl viologen reductase activity (1.45 *versus* 0.36 s^−1^), a 4.6-fold higher cytochrome *b*_5_ reductase activity (0.65 *versus* 0.14 s^−1^), a 4.5-fold higher Ngb reductase activity (0.90 *versus* 0.20 s^−1^), and a twofold higher trimethylamine-N-oxide (TMAO) reductase activity (0.41 *versus* 0.21 s^−1^). CO (50 μM) inhibits TMAO reduction by ∼90% suggesting an increased ET and reduction by the heme. Other reductase activities are relatively resistant to 50 μM CO. Increased rates of reduction demonstrate an increased ET from the flavin and a loss of the electron tunneling path directionality (44) and coupling control in the mutant.

Together these data support a role for the conserved LysF7 epsilon ammonium group in forming part of a switchable gate, or dynamic matrix, for ET that controls the rate, timing, and tunneling pathway for ET. Mistiming of heme reduction may also cause irreversible NO inhibition and a rapid inactivation; *i.e.*, a mistiming of ET creates the opportunity for NO to bind Fe^2+^ irreversibly during turnover.

## Discussion

Cooperative activation of the NOD function of the *E. coli* flavoHb by the substrates O_2_ and NO is explained by motions of the protein triggered by the gases and the product nitrate. Motions, although not directly observed, are inferred from the available x-ray crystal structures and from the effects of conserved amino acid substitutions on the steady-state and transient kinetics. Deficiencies and paradoxes of the rudimentary ping-pong model (3, 4) are resolved by an allosterically controlled ping-pong mechanism in which ET is activated by O_2_ and NO positioning (Fig. 3). A critical reevaluation of NADH-driven enzyme velocities (Table 4) and heme reduction rates demonstrates activation of ET by O_2_ and NO. Comparable changes in the rates of heme reduction in the absence of O_2_ and NO require approximately fourfold greater NADH concentrations (4), and thus more negative flavin redox potentials and a greater driving force. Furthermore, ET transfer rates measured by stopped flow (Fig. 9) reveal *on* and *off* states in resting state structures representative of steps in the dynamic switching mechanism. From the data in Table 1, Table 2, Table 3, Table 4, we can begin to estimate times and activation energies for the steps in catalysis and compute the effects of various mutations. For example, from the *V*_max_ values, we can calculate that the rate-limiting step of the WT enzyme requires 1.7 and 6.3 ms at 37 and 20 °C, respectively. The near diffusion-limited *V*_max_/*K*_3_ value of 2.3 × 10^9^ M^−1^ s^−1^ and the four- to eightfold larger *V*_max_/*K*_4_ value at 37 °C indicates that multiple NO collisions and the wiggling of G8, E15, E11 side chains within the ST are required for, and limit, ET activation and the NOD reaction. Applying the Arrhenius equation for *V*_max_ at the two temperatures, we can approximate an overall activation energy (*E*_a_) equal to 13.7 kcal/mol, an amount equivalent to that required to break three to four hydrogen bonds. The largest effect on the *E*_a_ is observed with the AlaE11 substitution with a calculated *E*_a_ of 24.6 kcal/mol and a 5- to 15-fold larger temperature-dependent *K*_T_ value further suggesting that LeuE11 movements due to NO collisions, and concerted motions of the CD loop, E and F-helices, and LysF7, form the largest energetic barrier, and also the rate-limiting step in the NOD mechanism. Following this analysis, we can also see that the forward rate constant for the trigger and switching mechanism, *k′*_T_, limits and equals the *V*_max_ value.

ET in flavoHb (*k*_ET_), on the other hand, has been reported to be ∼40-fold faster by time-resolved pulse radiolysis (6800 s^−1^) (39) than the maximal NADH-driven ET rate measured by stopped flow (160 s^−1^) (Fig. 9*B*) (4) indicating that ET *per se* is not rate limiting and suggesting experimental differences in the matrix, transfer distance, or FAD/FADH_2_ potential (E^0^ = < −0.15 V) (52). Furthermore, the HisE7 substitution may impair ET in the steady state (Fig. 8 and Table 4) and resting state (Fig. 9*B*, *line 4*) by strengthening bonding to the heme propionate in the ET gate structure and stabilizing the *off* state (Fig. 2*D*, *top*) and, moreover, by significantly decreasing the nominal −0.125 V heme mid-point potential (52). The E7 position is too distant for the histidine to coordinate with the iron. Nevertheless, the structures (Fig. 2*C*) together with spectral analysis (*unpublished results*) suggest a relaxed to tense motion in the heme corresponding to a high spin (*S* = 5/2) to low spin (*S* = 1/2) ferric iron transition (53) with a large calculable heme mid-point potential drop of ∼0.09 V that may account for decreased extents of heme reduction despite lower FAD/FADH_2_ potentials and a greater driving force (Fig. 9*B*). Furthermore, thermally fluctuating spin state mixtures in the resting state may account for the observed intermediate ET rates (Fig. 9*B*). Accordingly, the proposed conformational changes of the F-helix in the trigger step (*K*_T_) force a low to high spin transition and associated potential increase, besides switching the ET gate *on* (Figs. 2*D* and 3). Although matrix perturbations in outer sphere ET processes, as modeled for flavoHb in an *on* state (Fig. 2*D*, *bottom*) (38, 39), show greater effects on *k*_ET_ values than small differences in ΔE^0^ values with generally large 1-V reorganization energies (44), corrections to the nominal heme mid-point potential due to a mixed resting ferric iron spin state will also influence *k*_ET_ values following the Marcus electron-transfer equation (38, 39, 44).

The free energy released in the reaction of NO and O_2_^−^ forming peroxynitrite is 29 kcal/mol (54), and the reaction of NO with MbFe^3+^O_2_^−^ to form nitrate releases more than 30 kcal/mol (55). The Mb-catalyzed isomerization of peroxynitrite to nitrate occurs in the submillisecond time range (6, 56) indicating that *k*_is_ would not be rate limiting. In the model (Fig. 3), NO_3_^−^ release (*k*_p_) and efflux are coupled with CD loop unfurling–furling, and the rate constants thus equal *V*_max_. We suppose that the initial step in the exergonic NO dioxygenation reaction, as represented by *k'*_4_, where *k'*_4_ equals *V*_max_/*K*_4_ and *k'*_NOD_, ultimately provides the energy required to drive protein motions and support the catalytic cycle. Furthermore, by analogy, a 577-nm photon, bearing 49.6 kcal/mol, and absorbed by the flavoHb during photolysis of O_2_ or other bound ligands, would suffice to trigger large protein motions *via* LeuE11, TyrB10, and GlnE7 interactions and contribute to the microsecond-scale heterogeneity of ligand migration evidenced by the data in Table 5, Tables 5 and S5; and Figs. S3 and S4, and elsewhere (4, 5). In the photolysis of MbFe^2+^CO, most motion occurs in the nanosecond range, but CO-induced structural changes and hydration shell fluctuations, nevertheless, continue for microseconds (57, 58, 59, 60).

In the model, *K*_2_, not *K*_1_, and the apparent *K*_m_(O_2_) is only roughly approximated by *V*_max_/*k'*_slow_ (O_2_). *K*_1_ may also be further broken down to movement of O_2_ through the strictures between the docking sites S3, S2, and S1 with a relatively large 6 kcal/mol energy barrier estimated for the narrow stricture 2 controlling passage from S2 to S1 (38). Displacement of LeuE11 by nitrite, and presumably nitrate also, widens the clearance of A16-G16 in stricture 2 by 1.6 Å, and the clearance of E15-H12 in stricture 1 by 3.1 Å (Table S2). Indeed, the *K*_i(NO)_ values of 0.3 to 1.2 μM determined for NO competition with O_2_ for LT entry (Table 1), and also comparable *K*_i(CO)_ values of ∼1 μM (5), argue for the relatively small *K*_1_ value of 2 μM applied throughout in modeling the kinetics, and a facile entry and passage of O_2_. Variations in the number of O_2_ molecules in the LT with [O_2_] may explain the two- to threefold range in *K*_i(NO)_ values estimated for the WT flavoHb.

A surprising outcome or prediction of the allosteric model is that a paramagnetic O_2_ molecule, with an induced dipole, interacts magnetically with the paramagnetic ferric iron atom in displacing LeuE11 and, moreover, that TyrB10 and GlnE7, as well as the G-helix dipole, affect this interaction. Indeed, the Fe^3+^ and O_2_ interactions are relatively weak given the large micromolar *K*_2_ values (Tables 1 and 3) relative to the corresponding nanomolar *K*_d_ values for Fe^2+^O_2_ (Table 5). The strictured and segmented LT further suggests potential mechanisms for the controlled storage and influx of O_2_ in the NOD function of flavoHb and roles for similar tunnels in other globin-NODs including HbN, the alpha subunit of HbA (18, 61), and Cygb (10, 17, 36, 37, 50). The failure of O_2_ to compete with NO suggests strong discrimination against O_2_ entry by the ST. In the *S. cerevisiae* flavoHb ferric-nitrite structure, water molecules hydrogen bonded to GlyH9 and ProG6 peptide carbonyls guard the ST entryway. Nonpolar O_2_ does not readily penetrate hydration shells on protein surfaces (62), and some protein sites bind water more tightly than others (63). Polar NO may displace the polar water molecule(s) and enter, whereas O_2_ may not. In the case of thermoglobin (64) (Table S3), a putative NOD, the unique charged LysG9 and GluH9 pair may better serve the NO gating function at the hydrogen bond–breaking 95 °C of the archebacterial existence. The failure of ubiquitous and abundant N_2_, the carrier gas, to compete with O_2_ for LT entry and inhibit the NOD function also requires explanation. The greater than 80-fold selectivity for O_2_ over N_2_ apparent in the steady-state data (Fig. 1*A*) may be achieved by a discriminator for the inducible lateral O_2_ dipole. The diametric PheG20 phenyl group and ThrA12 O-atoms within stricture 3 (Fig. 2*B* and Table S1) may serve this function by conjugating and repelling π∗/π molecular orbitals thus allowing O_2_ passage, but excluding entry of the less polarizable N_2_ molecule.

The model also predicts that motions in the LysE7 epsilon ammonium group, triggered by NO motions and passage by the LeuE11 barrier in the ST, ultimately control the *on* and *off* states and tunneling path for ET, and, furthermore, that GlnE7 and GlnE2 movements participate in the switching mechanism as evidenced by the strong inhibitory effect of the HisE7 and E2 substitutions on ET (Table 4 and Figs. 8 and 9*B*). We suppose that a small *K*_T_ value, and a strong trigger, will favor the *on* position and that larger *K*_T_ values, like that shown by the HisE7 variant, will favor the *off* position in the resting state. Further exploration of the effects of mutations, and tunneling matrix modifiers or dopants, on ET rates in flavoHbs should be illuminating provided that the fine structure of the switch mechanism can be mirrored, and the FAD/FADH_2_ potential controlled, in the resting state(s). Furthermore, the model and Fig. S1 suggest that NO_3_^−^, instead of chloride, normally binds and stabilizes the unfurled *off* configuration of the CD loop. Moreover, CD loop furling–unfurling may play an unrecognized role in the channeling of nitrate by ThrE2 in HbN-NOD (34) and the channeling of other anions (65, 66) by various globins.

The data and model can also be used to determine the physiological limits of the allosteric NOD mechanism. The data in Figure 1*C* demonstrate kinetics consistent with the mechanism at a physiologically relevant 8 μM O_2_ with a velocity of 8 s^−1^ at 2 μM NO. Applying Equation 3 and the constants in Table 1, we can calculate that at 0.4 μM O_2_ and 1 μM NO, the velocity of the allosteric NOD activity approximates the *V*_max_ of 0.1 s^−1^ for Fe^2+^NO reduction (4, 5). A similar comparative analysis can be done to evaluate the functional significance of the bimolecular reaction of O_2_ with Fe^2+^NO/Fe^3+^NO^−^ (11), but the dependence of the reaction on [O_2_], and a small rate constant, make the reaction inconsequential (2). Consistent with this analysis, flavoHb fully protects *E. coli* growth under an atmosphere containing 5 μM O_2_ and 0.5 μM NO (67) and shows insignificant NO metabolic activity in anaerobic *E. coli* (68).

Finally, the aggregate data and model suggest that all globins functioning as NODs have evolved allosteric mechanisms for controlling gas movements (69), timing ET (10), expelling nitrate, and averting irreversible NO inhibition and a reductive inactivation. Further investigations of ET in flavoHb promise to increase our understanding of conformationally gated ET, the NOD mechanism of globins, and the evolutionary origins of the relaxed-tense heme transitions for allosteric cooperativity in O_2_ binding by O_2_-transporting Hbs (53).

## Experimental procedures

### Reagents

Reagents were purchased from Sigma-Aldrich Chemical Co (St Louis, MO, USA) unless otherwise stated. DNA primers were synthesized at the University of Cincinnati Medical Center DNA facility. Praxair (Bethlehem, PA, USA) was the supplier of 99.993% O_2_, 99.999% CO, and 99.98% N_2_. Bovine liver catalase (260,000 U/ml) was obtained from Roche Applied Science. Recombinant rat cytochrome *b*_5_ (soluble form) and murine Ngb were expressed in *E. coli* and purified essentially as described (10, 70). NADH purity (≥95%) was assessed by the ratio of the 340 to 260 nm absorbance with a value of 0.437 equal to 100% reduced.

### Construction of FlavoHb variants

pUC19*hmp* was constructed by ligating the EcoR1-BamH1 fragment from pAlter*hmp* (4) into pUC19 (71). Substitution mutants were constructed using the Altered Sites II mutagenesis system (Promega) with pAlter*hmp* as the template and the corresponding sense primers with codon changes shown in bold face type:

LeuG8, 5′-CAGTACAACATC**CTC**GGTGAACACCTG-3′;

AlaG8, 5′-CAGTACAACATC**GCC**GGTGAACACCTG-3′;

TrpE11, 5′-GATCAACGTGAAGCC**TGG**TTTAACGCTATTGCC-3′;

AlaE11, 5′-GATCAACGTGAAGCC**GCG**TTTAACGCTATTGCC-3′;

AsnE2, 5′-TTTAACATGAGTAAC**AAT**CGTAATGGCGATCAA-3′;

LeuE2, 5′-TTTAACATGAGTAAC**CTG**CGTAATGGCGATCAA-3′;

HisE2, 5′-TTTAACATGAGTAAC**CAT**CGTAATGGCGATCAA-3′;

GluE2, 5′-TTTAACATGAGTAAC**GAG**CGTAATGGCGATCAA-3′;

PheB10, 5′-CGCCCATTTC**TTC**GACCGTATGTT-3′;

LeuE7, 5′-GTAATGGCGAT**CTA**CGTGAAGCCC-3′;

GluE7, 5′-CGTAATGGCGAT**GAA**CGTGAAGCC-3′;

HisE7, 5′-TAATGGCGAT**CAC**CGTGAAGCCCTG-3′;

AsnF7 was constructed by PCR mutagenesis using pUC19*hmp* as the template with the sense primer 5′-CGGTAGAAAAAATCGCGCAG**AAT**CACACCAGCTT-3′ and the antisense primer 5′-CTGCGCGATTTTTTCTACCGCTGGCAGCAG-3′. All constructs were verified by DNA sequencing, and agarose gel purified NdeI-BamHI fragments of the pAlterNOD variant constructs were ligated into the NdeI-BamHI digested pUC19*hmp*. Engineered pUC19NOD variant constructs were transferred to the *hmp* deletion strain AG103 (68) for expression.

#### Expression and purification of NODs

Cultures of AG103 bearing pUC19*hmp*, and engineered variants thereof, were grown microaerobically in a phosphate-buffered medium containing yeast extract, tryptone, glucose, nitrate, hemin, and ampicillin and were harvested, purified, and analyzed as previously described (4). Batches of 1-l cultures were grown microaerobically in 2-l flasks by a slow gyrorotatory motion and were harvested after 16 h. Isolated flavoHbs contained between 0.6 and 0.9 mol fraction of heme and between 0.3 and 0.9 mol fraction of FAD. FlavoHbs were reconstituted with heme and FAD (4,72).

#### Assay of NOD and other flavoHb activities

NOD activity was measured amperometrically in 100 mM phosphate buffer, pH 7.0, containing 0.3 mM EDTA, 100 μg/ml bovine serum albumin (BSA) (Fraction V), 100 μM NADH, 0.1 μM FAD with NO, O_2_, and N_2_ mixtures added as described (4, 72). NADH oxidase activity was followed spectrophotometrically at 340 nm in a 1.0-ml cuvette containing 90 mM Tris chloride buffer, pH 8.2, 100 μM NADH, 200 μM O_2_, 0.1 μM FAD, 100 μg/ml BSA, and 0.3 mM EDTA and incubated at 37 °C. Cytochrome *b*_5_ reductase activity was assayed at 37 °C by following the rate of absorbance increase at 555 nm in a 1.0-ml reaction buffered with 90 mM Tris chloride, pH 8.2, and containing 10 μM ferric cytochrome *b*_5_, 200 μM O_2_, 20 μM NADH, 0.1 μM FAD, 100 μg/ml BSA, and 0.3 mM EDTA. TMAO reductase activity was assayed by following the absorbance decrease at 340 nm in a 1.0-ml anaerobic reaction containing 12 U *Aspergillus niger* glucose oxidase, 5 mM glucose, 260 U catalase, 400 mM TMAO, 100 μM NADH, 0.1 μM FAD, 100 μg/ml BSA, and 0.3 mM EDTA in 90 mM Tris chloride pH 8.2 and incubated at 37 °C. Ngb reductase activity was assayed by following the absorbance increase at 424 nm in a 1.0-ml anaerobic reaction containing 12 U glucose oxidase, 5 mM glucose, 260 U catalase, 5 μM Ngb, 50 μM NADH, 0.1 μM FAD, 100 μg/ml BSA, and 0.3 mM EDTA in 90 mM Tris chloride buffer, pH 8.2, and incubated at 37 °C. Methyl viologen reductase activity was assayed by following the formation of the blue cation radical at 603 nm and by applying an extinction coefficient of 13,300 M^−1^ cm^−1^ in a 1.0-ml anaerobic reaction buffered with 10 mM potassium phosphate, pH 7.0, and containing 12 U glucose oxidase, 5 mM glucose, 260 U catalase, 100 μM NADH, 0.1 μM FAD, 100 μg/ml BSA, 0.3 mM EDTA, and 200 mM sodium chloride and incubated at 37 °C. NO reductase activity was assayed amperometrically at 37 °C in a 1.0-ml anaerobic reaction containing 2 μM NO, 100 μM NADH, 0.1 μM FAD, 12 U glucose oxidase, 5 mM glucose, 260 U catalase, 100 μg/ml BSA, and 0.3 mM EDTA buffered with 10 mM potassium phosphate pH 7.0. CO effects were tested at a final concentration of 50 μM as diluted from a 1 mM stock in water. All activities are reported relative to the heme content of flavoHb.

#### Model for NOD kinetics and fitting

Rate constants were estimated from the model kinetic equation using Python programming with successive approximations to optimize fits with varying NO and O_2_ concentrations. *V*_max_, *K*_1_, *K*_2_, *K*_T_, and *K*_ii(NO)_ values were treated as independent variables for a given flavoHb while allowing *K*_3_, *K*_4_, and *K*_i(NO)_ to vary over limited ranges with varying O_2_ concentration. *K*_ii(NO)_ was estimated from NO inhibition at saturating O_2_ concentrations. *K*_i(O2)_ and *K*_a(O2)_ were included as independent variables in order to model the AlaE11 mutant data.

#### Reduction and ligand binding kinetics

FAD and heme reduction were monitored by following the changes in flavoHb absorbance at 460 and 430 nm, respectively, with stopped-flow rapid mixing of 50 μM NADH and 10 μM flavoHb in N_2_-purged buffer (4). O_2_, NO, and CO association rate constants were determined by photolyzing 50 μM of the respective ferrous flavoHb species with a 300-ns pulse from a 577-nm laser and by spectrophotometrically following ligand reassociation in O_2_-, NO-, or CO-saturated buffer unless otherwise indicated (4, 5). Binding of O_2_ or CO was measured by the change of absorbance at 436 nm by applying the bound *versus* unbound difference extinction coefficients of 0.046 and 0.053 μM^−1^ cm^−1^, respectively. Binding of NO was measured by the change of absorbance at 422 nm by applying a difference extinction coefficient of 0.040 μM^−1^ cm^−1^. O_2_ and CO dissociation rate constants were determined by stopped-flow rapid mixing with either CO or NO displacement (4, 5). The cuvette path length was 1.0 cm. All reactions were at 20 °C in filtered 100 mM sodium phosphate buffer, pH 7.0, containing 0.3 mM EDTA. Catalase (1000 U/ml) was included in all reactions containing O_2_ to scavenge H_2_O_2_ produced by the NADH oxidase activity of flavoHb and to regenerate O_2_.

#### Data analysis

Data are representative of two or more trials. Structures were modeled and analyzed using Jmol version 14.2.15.

## Data availability

All data are contained within the manuscript.

## Conflict of interest

The authors declare that they have no conflicts of interest with the contents of this article.
