# Supplementary material for: Allostery in the nitric oxide dioxygenase mechanism of flavohemoglobin
Source: J Biol Chem. 2020 Dec 17;296:100186. doi: 10.1074/jbc.RA120.016637 (PMC7948479; doi:10.1074/jbc.RA120.016637)
Supplement: Supplementary Figures and Tables [file mmc1.pdf]

Supporting Information

Allostery in the nitric-oxide dioxygenase mechanism of flavohemoglobin

Anne M. Gardner and Paul R. Gardner

Table of Contents

Item	Description	Page
Table S1	Conservation of Long Tunnel and pocket residues in flavohemoglobins (flavoHbs) and single domain globins	S-2
Table S2	Long Tunnel stricture dimensions	S-3
Table S3	Conservation of Short Tunnel residues in flavoHbs and single domain globins	S-4
Table S4	Short Tunnel static dimensions	S-5
Table S5	CO and O_2 binding to ferrous flavoHb-NODs	S-6
Figure S1	Model for CD loop furling and unfurling	S-7
Figure S2	CD loop conformations in the dimeric *Ralstonia eutropha* flavoHb-econazole structure	S-8
Figure S3	Biphasic ligand rebinding kinetics of WT, B10 and E7 mutant flavoHbs	S-9
Figure S4	Biphasic O_2 rebinding kinetics of WT flavoHb	S-10
Figure S5	H_2O_2 sensitivity of the flavoHb-NOD	S-11
References		S-12

Table S1. Conservation of Long Tunnel and pocket residues in flavoHbs and single domain globins

Position	*Ec*	*Sc*	*Re*	*Vs*	*Aa*
Surface					
A11	Ala^{12}	Ala^{12}	Ala^{12}	Ala^{12}	**Ser^{12}**
Stricture 3					
A12	Thr^{13}	Thr^{13}	Thr^{13}	Thr^{13}	Thr^{13}
A15	Leu^{16}	Val^{16}	Val^{16}	Val^{16}	Leu^{16}
G20	**Phe^{110}**	Leu^{110}	Leu^{110}	Leu^{110}	Leu^{107}
H4	Val^{116}	Ile^{118}	Ile^{118}	Ile^{118}	Val^{113}
Stricture 2					
A16	Leu^{17}	Leu^{17}	Leu^{17}	Leu^{17}	Leu^{17}
B5	**Leu^{24}**	Ile^{24}	Ile^{24}	Ile^{24}	Ile^{24}
G16	**Leu^{106}**	Ile^{106}	Ile^{106}	Ile^{106}	Ile^{103}
H8	Trp^{120}	Trp^{122}	Trp^{122}	Trp^{122}	Trp^{117}
Stricture 1					
E15	Ile^{61}	Val^{61}	Val^{61}	Val^{61}	Ile^{58}
G12	Leu^{102}	Leu^{102}	Leu^{102}	Leu^{102}	Leu^{99}
H12	Tyr^{124}	Tyr^{126}	Tyr^{126}	Tyr^{126}	Tyr^{121}
H15	Leu^{127}	Ile^{129}	Leu^{129}	Ile^{129}	Leu^{124}
Pocket					
B9	Phe^{28}	Phe^{28}	Phe^{28}	Phe^{28}	**Met^{28}**
B10	Tyr^{29}	Tyr^{29}	Tyr^{29}	Tyr^{29}	Tyr^{29}
E7	Gln^{53}	Gln^{53}	Gln^{53}	Gln^{53}	Gln^{50}
E11	Leu^{57}	Leu^{57}	Leu^{57}	Leu^{57}	Leu^{54}
E12	**Phe^{58}**	Ala^{58}	Ala^{58}	Ala^{58}	Ala^{55}

Ec, *Escherichia coli*; *Sc*, *Saccharomyces cerevisiae*; *Re*, *Ralstonia eutropha*; *Vs*, *Vitreoscilla stercoraria; Aa, Aquifex aeolicus*

Table S2. Long Tunnel stricture dimensions

Stricture Dimension	*Ec* ferric (1GVH)	*Sc* ferric-NO_2^- (4G1V)	*Sc* ferric-econ (4G1B)	*Re* ferric-econ (3OZV) /micon (3OZU)	*Vs* ferric-N_3^- (2VHB)
Stricture 3					
G20-H4	7.6	5.2	6.9	5.8/5.7	6.1
H4-A12	5.0	4.1	4.5	3.7/3.6	4.7
A12-A15	4.9	5.6	6.1	6.0/5.4	4.8
A15-G20	4.1	4.4	4.3	4.1/6.0	4.7
static clearance	6.8 *A15-H4*	4.6 *A12-G20*	6.2 *A12-G20*	6.4/6.3 *A15-H4*	5.6 *A12-G20*
Stricture 2					
G16-H8	5.5	5.1	5.8	6.5/5.8	4.6
H8-A16	3.8	4.8	4.5	4.3/4.6	4.7
A16-B5	4.3	4.6	4.7	5.1/5.0	4.2
B5-G16	4.8	5.3	5.7	5.2/5.0	6.0
static clearance	3.7 *A16-G16*	5.3 *A16-G16*	4.7 *A16-G16*	4.8/4.5 *A16-G16*	4.0 *A16-G16*
Stricture 1					
G12-H12	4.1	3.9	3.8	4.6/4.0	4.0
H12-H15	4.9	5.8	5.9	6.3/6.7	6.1
H15-E15	**4.8**	7.5	6.9	8.1/7.9	**4.2**
E15-G12	**4.2**	4.3	5.2	7.5/7.6	**3.8**
static clearance	**4.2** ***E15-H12***	7.3 *E15-H12*	**7.9** ***E15-H12***	10.1/10.0 *E15-H12*	**4.6** ***E15-H12***

Values are given in Å for the minimal atomic distances excluding H-atoms for residues at positions in the *Ec* (1); *Sc* (2); *Re* (3); and *Vs* (4) globin X-ray crystal structures with the PDB codes in parentheses. Econ; econazole, micon; miconazole.

Table S3. Conservation of Short Tunnel residues in flavoHbs and single domain globins

Position	*Ec*	*Sc*	*Re*	*Vs*	*Aa*
G9	Gly^{99}	Gly^{99}	Gly^{99}	Gly^{99}	**Lys^{96}**
H9	Gly^{121}	Gly^{123}	Ala^{123}	Gly^{123}	**Glu^{118}**
G13	Leu^{103}	Leu^{103}	Leu^{103}	Leu^{103}	Leu^{100}
H12	Tyr^{124}	Tyr^{126}	Tyr^{126}	Tyr^{126}	Tyr^{121}
G8	Val^{98}	Val^{98}	Val^{98}	Val^{98}	Val^{95}
E15	Ile^{61}	Val^{61}	Val^{61}	Val^{61}	Ile^{58}
E11	Leu^{57}	Leu^{57}	Leu^{57}	Leu^{57}	Leu^{54}

Table S4. Short Tunnel static dimensions

	Ec ferric (1GVH)	*Sc* ferric-NO_2^- (4G1V)	*Sc* ferric-econ (4G1B)	*Re* ferric-econ (3OZV)/ -micon (3OZU)	*Vs* ferric (3TM3)	*Vs* ferric-N_3^- (2VHB)
Entrance						
G9-H9	7.2	7.3	6.9	8.2/7.8	7.2, 7.0	7.4
G13-H12	6.3	6.5	6.8	6.0/5.7	5.6, 4.7	5.3
Gate						
G8-E15	3.7	**8.0**	**9.2**	**12.1/11.7**	4.7, 5.7	6.1
G8-E11	5.4	6.3	**9.5**	**10.1/9.8**	4.7, 4.1	4.4
G8-Fe	7.8	6.3	7.1	6.2/6.3	7.0, 6.6	6.6
Trigger						
E11-Fe	3.5	**5.7**	**11.7**	**9.9/9.3**	3.7, 3.7	4.6

Values are given in Å for the minimal atomic distances excluding H-atoms for residues at positions in the *Ec* (1); *Sc* (2); *Re* (3); and *Vs* (4,5) globin X-ray crystal structures with the PDB codes in parentheses.

Table S5. CO and O_2 binding to ferrous flavoHb-NODs

Photolysis and rebinding reactions were carried out with 100 µM CO in the presence of 0.6 mM N_2 or 1 mM O_2 as described under Experimental Procedures. Φ_{dis} , photodissociation; τ_T , transition time.

**Ligand Pair**	Φ_{dis}	***k'* fast**	τ_T	***k'* slow**
	%	$\mu M^{-1}\ s^{-1}$	μs	$\mu M^{-1}\ s^{-1}$
**Fe^{2+} CO**				
WT	15	29 (56%)	500 ± 100	2.7 (44%)
PheB10	12	38 (58%)	400 ± 200	2.6 (42%)
LeuE7	11	9.9 (38%)	2500 ± 500	1.9 (62%)
HisE7	14	16 (50%)	500 ± 100	1.3 (50%)
**Fe^{2+} O_2**				
WT	4	29 (64%)	70 ± 10	1.9 (36%)
PheB10	6	111 (61%)	15 ± 5	6.2 (39%)
LeuE7	8	19 (80%)	125 ± 25	1.8 (20%)
HisE7	3	66 (30%)	8 ± 3	7.2 (70%)

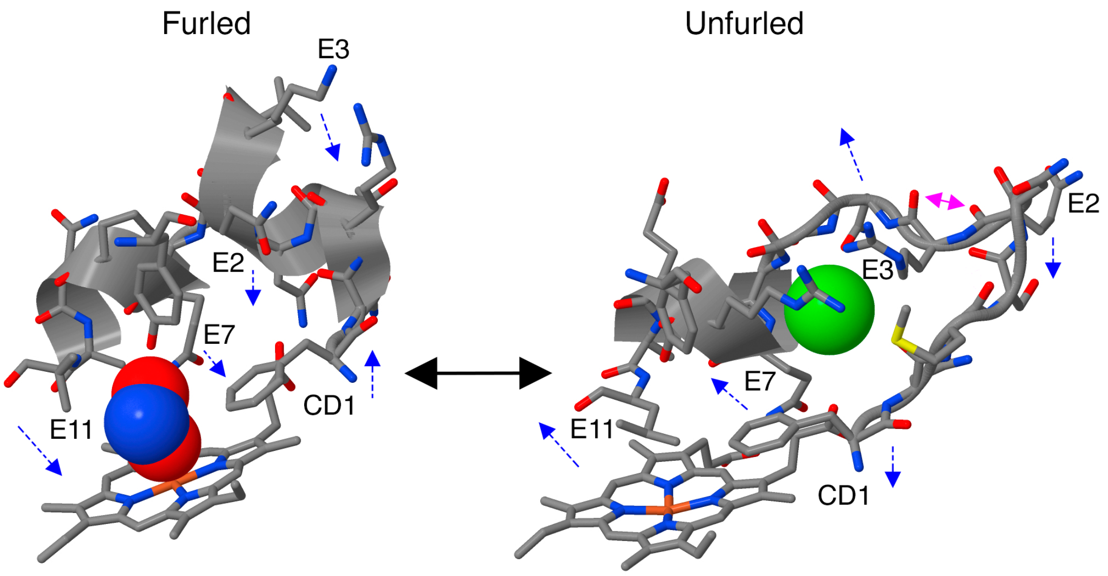

Figure S1. Model for CD loop furling and unfurling. CD loop of ferric-nitrite flavoHb in the furled state (*left*) shown interconverting with the ferric flavoHb CD loop in the unfurled state (*right*) with heme ligand driven torsional movements of the E11, E7, E3, E2, and CD1 residues (*blue arrows*). Chloride ion (*green*) shown trapped in the putative nitrate anion hole. Repulsion between the E2 and E3 peptide carbonyls shown in the unfurled state (*magenta arrow*). *E. coli* ferric chloride flavoHb and *S. cerevisae* ferric nitrite flavoHb X-ray structures (PDB ID: 1GVH (1) and 4G1V (2), respectively) were used to model the respective unfurled and furled states.

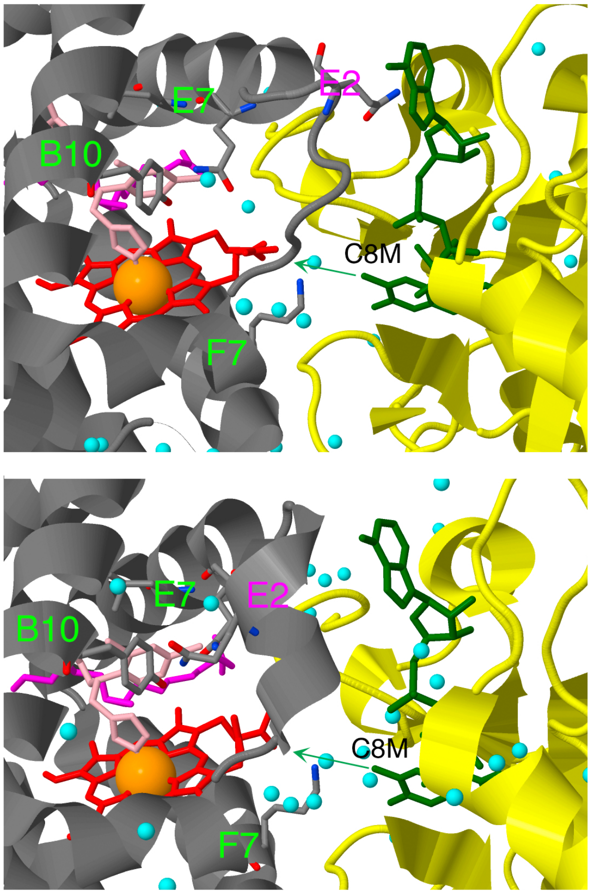

Figure S2. CD loop conformations in the dimeric *Ralstonia eutropha* flavoHb-econazole structure (PDB ID: 3OZV) (3). (*top*) The A subunit in the *off* position with the GlnE2 side chain extended toward the adenine ring of FAD and the CD loop in an extended coil. (*bottom*) The B subunit in the *on* position with the GlnE2 side chain in the distal pocket with the CD loop adopting a short α-helix conformation. Residues 191-206 have been removed from the structure foreground to improve visualization. The color code is Hb domain (*grey*), reductase domain (*yellow*), heme (*red*), iron (*orange*), FAD (green), water (*aqua*), amino acid side chains (CPK), econazole (*pink*), and fatty acid (*magenta*).

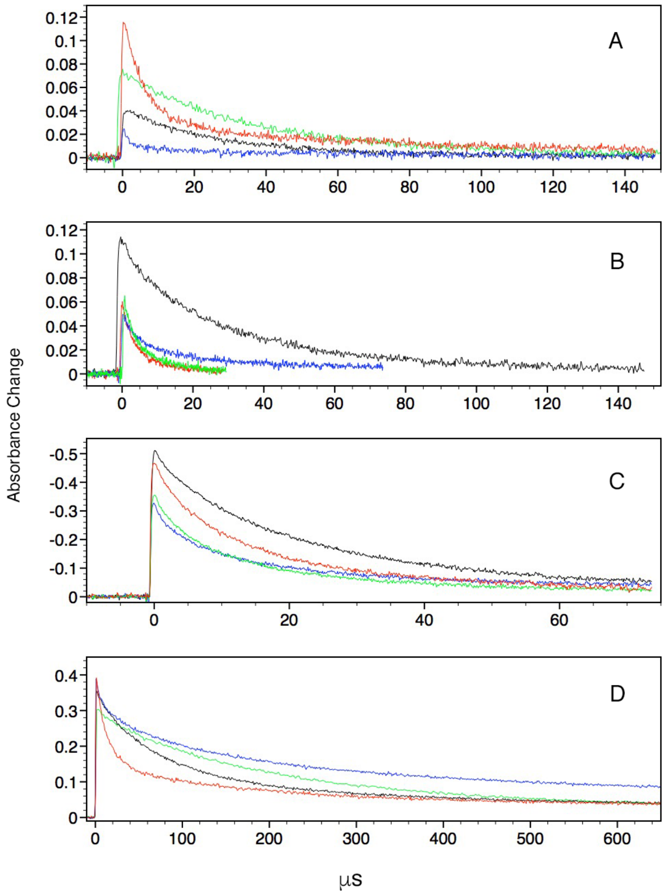

Figure S3. Biphasic ligand rebinding kinetics of WT, B10 and E7 flavoHb variants. WT (*black*), PheB10 (*red*), LeuE7 (*green*), and HisE7 (*blue*) flavoHbs in the (*A*) ferrous O_2, (*B*) ferrous NO, (*C*) ferric NO, (*D*) ferrous CO forms were photolyzed, and ligand rebinding was followed spectrophotometrically as described under Experimental Procedures.

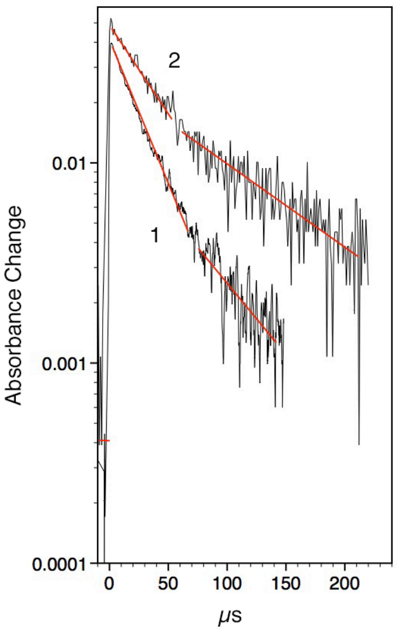

Figure S4. Biphasic O_2 rebinding kinetics of WT flavoHb. Rebinding of O_2 to the WT ferrous flavoHb was measured in the presence of 1140 μM O_2 (*line 1*) or 250 μM O_2 balanced with N_2 (*line 2*).

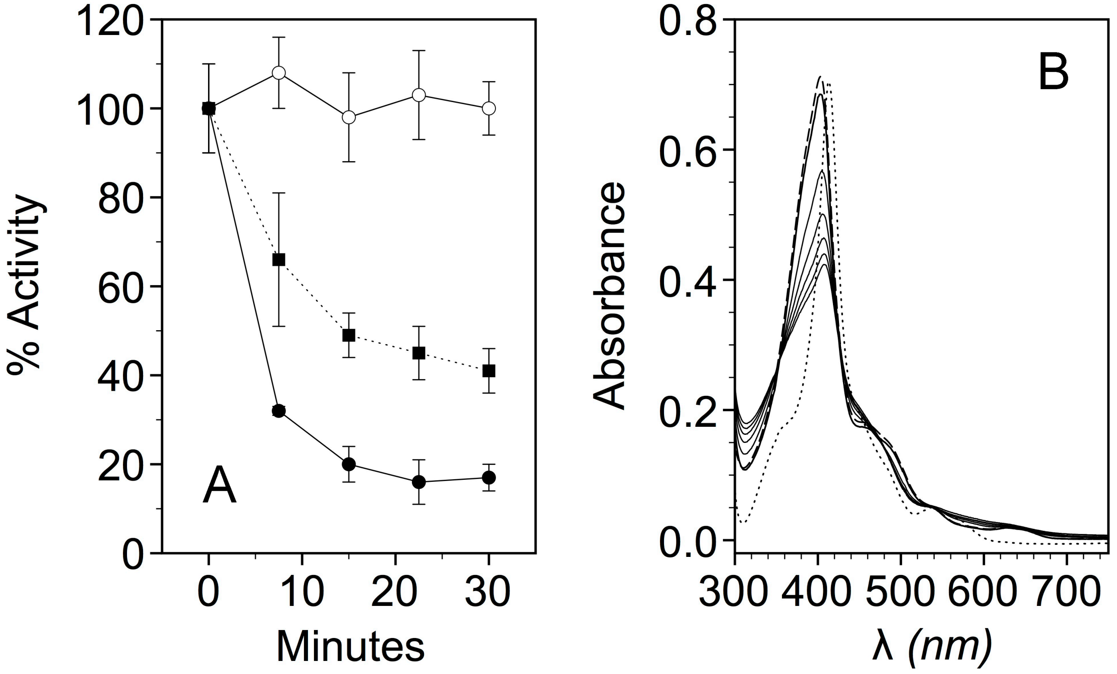

Figure S5. H_2O_2 sensitivity of the flavoHb-NOD. NOD activity of flavoHb is rapidly lost upon exposure to H_2O_2 (panel *A*), and heme absorbance decreases with H_2O_2 exposure (panel *B*). The peroxidizable substrate ascorbate and the heme ligand miconazole (6) afford protection thus demonstrating a role for Fe^{3+}-OOH in heme destruction or release. (*A*) FlavoHb (0.1 µM heme) was exposed to 0 (○), 50 (■) or 200 µM H_2O_2 (●). Catalase (1000 U/mL) was added at various times to terminate reactions, and NOD activity was measured as described under Experimental Procedures. (*B*) Ferric flavoHb (5 µM heme) was exposed to 200 µM H_2O_2, and spectra were recorded at 2 min intervals (*solid lines*) or after a 10 min exposure in the presence of 2 mM ascorbate (*dashed line*) or 10 µM miconazole (*dotted line*). Reactions were at 37°C in 50 mM potassium phosphate buffer, pH 7.8, containing 0.1 mM EDTA. Error bars represent the S.D. of three trials.

References

1. Ilari, A., Bonamore, A., Farina, A., Johnson, K. A., and Boffi, A. (2002) The X-ray structure of ferric *Escherichia coli* flavohemoglobin reveals an unexpected geometry of the distal heme pocket. *J. Biol. Chem.* **277**, 23725-23732
2. El Hammi, E., Warkentin, E., Demmer, U., Marzouki, N. M., Ermler, U., and Baciou, L. (2012) Active site analysis of yeast flavohemoglobin based on its structure with a small ligand or econazole. *FEBS J.* **279**, 4565-4575
3. El Hammi, E., Warkentin, E., Demmer, U., Limam, F., Marzouki, N. M., Ermler, U., and Baciou, L. (2011) Structure of *Ralstonia eutropha* flavohemoglobin in complex with three antibiotic azole compounds. *Biochemistry* **50**, 1255-1264
4. Tarricone, C., Galizzi, A., Coda, A., Ascenzi, P., and Bolognesi, M. (1997) Unusual structure of the oxygen-binding site in the dimeric bacterial hemoglobin from *Vitreoscilla* sp. *Structure* **5**, 497-507
5. Ratakonda, S., Anand, A., K., D., Stark, B. C., and Howard, A. J. (2013) Crystallographic structure determination of B10 mutants of *Vitreoscilla* hemoglobin: Role of Tyr29 (B10) in the structure of the ligand-binding site. *Acta Crystallogr. Sect. F Struct. Biol. Cryst. Commun.* **69**, 215-222
6. Helmick, R. A., Fletcher, A. E., Gardner, A. M., Gessner, C. R., Hvitved, A. N., Gustin, M. C., and Gardner, P. R. (2005) Imidazole antibiotics inhibit the nitric oxide dioxygenase function of microbial flavohemoglobin. *Antimicrob. Agents Chemother.* **49**, 1837-1843
